# Genetic signatures of gene flow and malaria-driven natural selection in sub-Saharan populations of the "endemic Burkitt Lymphoma belt"

**DOI:** 10.1371/journal.pgen.1008027

**Published:** 2019-03-08

**Authors:** Mateus H. Gouveia, Andrew W. Bergen, Victor Borda, Kelly Nunes, Thiago P. Leal, Martin D. Ogwang, Edward D. Yeboah, James E. Mensah, Tobias Kinyera, Isaac Otim, Hadijah Nabalende, Ismail D. Legason, Sununguko Wata Mpoloka, Gaonyadiwe George Mokone, Patrick Kerchan, Kishor Bhatia, Steven J. Reynolds, Richard B. Birtwum, Andrew A. Adjei, Yao Tettey, Evelyn Tay, Robert Hoover, Ruth M. Pfeiffer, Robert J. Biggar, James J. Goedert, Ludmila Prokunina-Olsson, Michael Dean, Meredith Yeager, M. Fernanda Lima-Costa, Ann W. Hsing, Sarah A. Tishkoff, Stephen J. Chanock, Eduardo Tarazona-Santos, Sam M. Mbulaiteye

**Affiliations:** 1 Instituto de Pesquisa René Rachou, Fundação Oswaldo Cruz, Belo Horizonte, Minas Gerais, Brazil; 2 Departamento de Biologia Geral, Instituto de Ciências Biológicas, Universidade Federal de Minas Gerais, Belo Horizonte, Minas Gerais, Brazil; 3 Center for Research on Genomics & Global Health, National Institutes of Health, US Department of Health and Human Services, Bethesda, Maryland, United States of America; 4 Division of Cancer Epidemiology and Genetics, National Cancer Institute, National Institutes of Health, US Department of Health and Human Services, Bethesda, Maryland, United States of America; 5 Departamento de Genética e Biologia Evolutiva, Instituto de Biociências, Universidade de São Paulo, São Paulo, Brazil; 6 Department of Statistics, Universidade Federal de Minas Gerais, Belo Horizonte, Minas Gerais, Brazil; 7 EMBLEM Study, African Field Epidemiology Network, Kampala, Uganda; 8 University of Ghana Medical School, Accra, Ghana; 9 Department of Biological Sciences, University of Botswana, Gaborone, Botswana; 10 Department of Biomedical Sciences, University of Botswana School of Medicine, Gaborone, Botswana; 11 Division of Intramural Research, National Institute of Allergy and Infectious Diseases, National Institutes of Health, US Department of Health and Human Services, Bethesda, Maryland, United States of America; 12 Laboratory of Translational Genomics, Division of Cancer Epidemiology and Genetics, National Cancer Institute, National Institutes of Health, US Department of Health and Human Services, Bethesda, Maryland, United States of America; 13 Cancer Genomics Research Laboratory, Leidos Biomedical Research, Frederick National Laboratory for Cancer Research, US Department of Health and Human Services, Frederick, Maryland, United States of America; 14 Stanford Cancer Institute, Stanford University, Stanford, California, United States of America; 15 Department of Genetics and Biology, University of Pennsylvania, Philadelphia, United States of America; University of Michigan, UNITED STATES

## Abstract

Populations in sub-Saharan Africa have historically been exposed to intense selection from chronic infection with *falciparum* malaria. Interestingly, populations with the highest malaria intensity can be identified by the increased occurrence of endemic Burkitt Lymphoma (eBL), a pediatric cancer that affects populations with intense malaria exposure, in the so called “eBL belt” in sub-Saharan Africa. However, the effects of intense malaria exposure and sub-Saharan populations’ genetic histories remain poorly explored. To determine if historical migrations and intense malaria exposure have shaped the genetic composition of the eBL belt populations, we genotyped ~4.3 million SNPs in 1,708 individuals from Ghana and Northern Uganda, located on opposite sides of eBL belt and with ≥ 7 months/year of intense malaria exposure and published evidence of high incidence of BL. Among 35 Ghanaian tribes, we showed a predominantly West-Central African ancestry and genomic footprints of gene flow from Gambian and East African populations. In Uganda, the North West population showed a predominantly Nilotic ancestry, and the North Central population was a mixture of Nilotic and Southern Bantu ancestry, while the Southwest Ugandan population showed a predominant Southern Bantu ancestry. Our results support the hypothesis of diverse ancestral origins of the Ugandan, Kenyan and Tanzanian Great Lakes African populations, reflecting a confluence of Nilotic, Cushitic and Bantu migrations in the last 3000 years. Natural selection analyses suggest, for the first time, a strong positive selection signal in the *ATP2B4* gene (rs10900588) in Northern Ugandan populations. These findings provide important baseline genomic data to facilitate disease association studies, including of eBL, in eBL belt populations.

## Author summary

We present a genome-wide analyses of genetic structure, gene flow, and natural selection in Ghana and Northern Uganda populations, both residing in the Sub-Saharan eBL belt, a region with intense *falciparum* malaria transmission and high endemic Burkitt Lymphoma (eBL) incidence. These populations are from different ethnolinguistic groups and are located 2400 miles apart in sub-Saharan Africa. We characterized genetic composition of these populations in the context of 22 additional African populations and present evidence for gene flow events that occurred in the last 3000 years, possibly related to regional migrations in Western Africa and major migrations involving Nilotic, Cushitic, and Bantu groups. We identified in Northern Ugandans a strong signal of malaria-driven selection in the *ATP2B4* gene coding for a calcium transporter expressed in erythrocytes. Characterization of biological relationships between the *ATP2B4* gene and malaria may inform the investigation of complex genomic disease associations in eBL belt populations.

## Introduction

**Fig 1:**
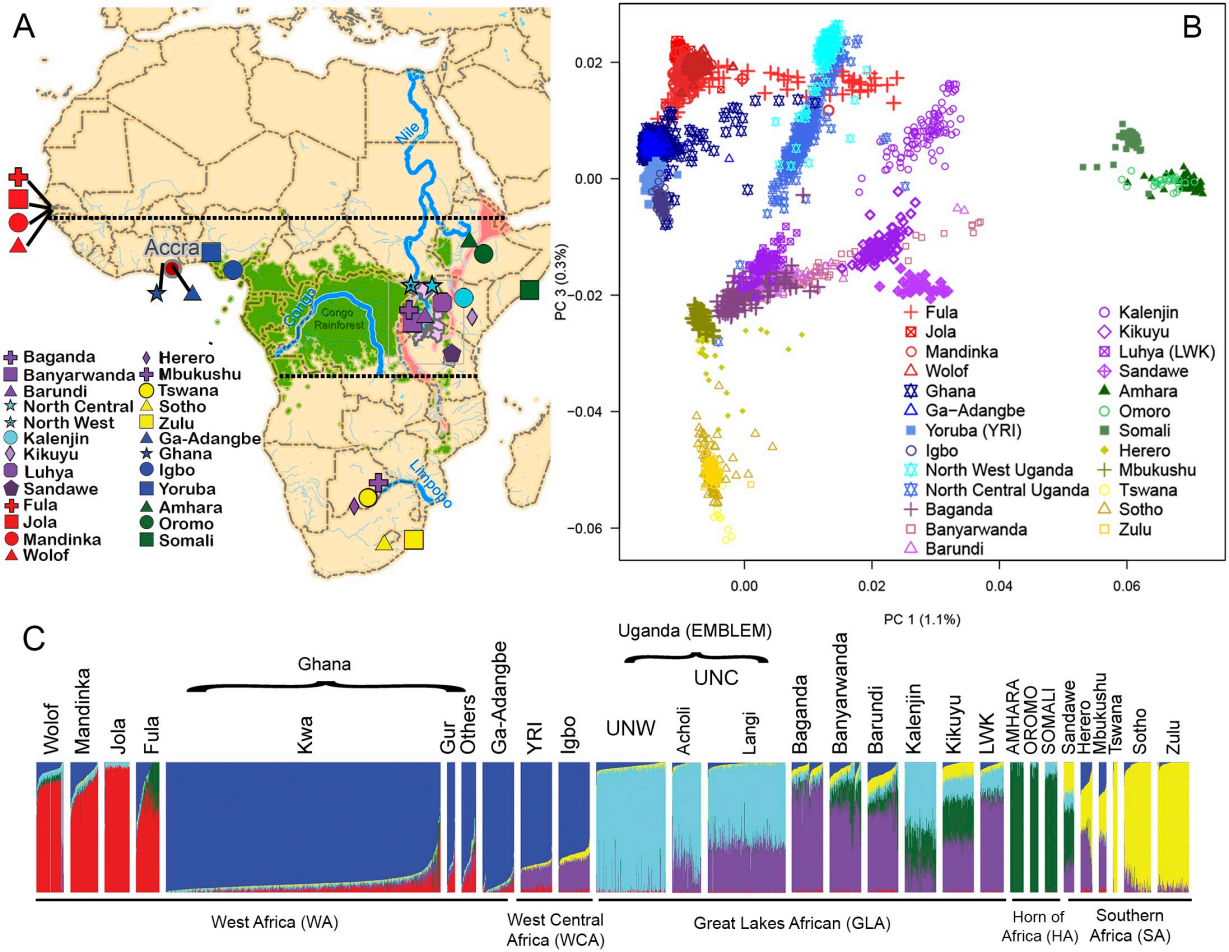
Populations studied in relation to major geographical barriers and analyses of population structure based on genotype data. (A) Map of Africa showing the geographical origin of the Pan-African populations used in the study comprising 22 populations from previous studies and three new populations in Ghana and the Uganda EMBLEM study (S1 Table). Horizontal dotted lines are the geographical extent of the endemic Burkitt lymphoma (eBL) belt (see S1 Fig and S1 Table for detailed information on Pan-African populations in the eBL belt). The map highlights major geographical features, such as the Congo rainforest (light green), major rivers and lakes, and the East and West African Rift valley systems (pink) that may have shaped migratory routes or constituted barriers to gene flow. (B) Principal Component Analysis (PCA) based on analysis of the genome-wide dataset of Uganda and Ghana integrated with 22 other pan-African populations. For better visualization, open symbols are used for the PCA plot and solid symbols are used in the map. The axes of the plot show the first and third principal components (See S10 Fig for other principal components). The PCA was repeated using similar number of individuals for each studied population (S9 Fig). (C) ADMIXTURE plot showing ancestry clusters in 28 populations from Africa (details in S1, S1A and S1B Table). The populations are listed left to right based on their geographical location in Africa from West to East and North to South. The colors represent different ancestral clusters, with K = 6 being the most likely number of clusters on admixture analysis (See S4 Fig). This ADMIXTURE analysis was repeated using similar number of individuals for each studied population (S6 Fig).

The endemic Burkitt Lymphoma (eBL) belt is a geographic area spanning 10°N-10°S and altitudes below 1500m above sea level (Fig 1A) in sub-Saharan Africa, where there is a high geographical correlation between malaria and eBL (an aggressive pediatric B-cell non-Hodgkin lymphoma). This correlation has led to the identification of malaria infection as a major driver of eBL [1][2], which was confirmed by the evidence that the sickle cell trait that protects against severe malaria [3] also protects against eBL [4]. Because eBL occurs in areas of sub-Saharan Africa [5] with stable intense *Plasmodium falciparum (Pf)* malaria (for 7–12 months in the year), eBL burden provides a novel way to identify populations under strong malaria selective pressure. *Pf* malaria is one of the most important selective pressures that have shaped the African genetic diversity [6], but there are limited reports on the combined effects of malaria-related natural selection and the demographic history of populations in the eBL belt.

The eBL belt was the scenario of several human migrations over the last 3000 years and archaeological and linguistic evidence have described the following historical events: in West Africa: (i) interaction between West and West-Central Africa [7], (ii) cultural interaction between the local kingdoms of West-Central Africa [8,9], and (iii) migrations across the Sahel that include the westward Nilotic expansion [7,10,11]. In East Africa: (iv) Eastern Cushitics migrated from the Horn of Africa [7] into the Great Lakes region ~3000 years ago, maintaining (v) interactions with Nilotic groups that migrated from Southern Sudan [10,11], and subsequently, (vi) with Bantu speakers from West-Central Africa who reached the Great Lakes region ~2000 years ago [7,12–15]. Moreover, malaria imposed an important evolutionary pressure well known for its effect on the genetic structure of affected populations, such as those that settled in the eBL belt.

Datasets representing African populations, such as those included in the 1000 Genomes Project [16], the African Genome Variation Project [17], the Tishkoff laboratory [18][11] and the H3Africa initiative [19][20], have provided an important baseline for genomic studies in Africa. However, due to the high genetic diversity among African populations, reference datasets should closely match populations in which specific scientific questions are explored. For example, the Nilotics in the Great Lakes region on Northern Uganda region, which experience high malaria intensity [21] and high eBL burden (S1 Table), have not been included in previous genomic studies [18].

To determine if the historical migrations described above (i-vi) and intense exposure to malaria have shaped the genetic composition in the eBL belt, we analyzed a new dataset of 945 Ghanaians and 568 Northern Ugandans in whom ~4.3 million single nucleotide polymorphisms (SNPs) were genotyped. These sub-Saharan Africa populations reside on opposite longitudes of the eBL belt (2400 miles apart) (Fig 1A), and are both exposed to high malaria pressure and have published evidence indicating a high eBL burden (S1 Table) [22].

## Results

### Study populations

Details of the study populations are given in S2 and S3 Tables. Briefly, the Ghanaian population included approximately 35 tribes, predominantly from the Kwa and Gur Niger-Congo language families (S2 Table). The Ugandan populations included approximately 17 tribes, predominantly of the Western Nilo-Saharan (Nilotic) language family (S3 Table). Because the Ugandan populations were recruited from opposite sides of the deep gorge of the East African Rift Valley, through which the Albertine Nile flows (Fig 1A) and this is a potential physical barrier to gene flow, we designated the populations descriptively as Uganda North West (UNW) for those recruited from the west side of the gorge and Uganda North Central (UNC) for those recruited from the east side of the gorge. We estimated the level of genetic relatedness of our dataset and excluded closely related individuals that may affect population-structure and natural selection analyses [23] (S1 Text and S2–S5 Figs).

### Population structure and gene flow dynamics in the eBL belt

Population structure was evaluated using a Pan-African genome-wide dataset (PA dataset, Methods) that included 1.3M SNPs genotyped in 3,102 individuals, including 1,513 from the combined UNW, UNC, and National Cancer Institute (NCI) Ghana datasets, and 1,589 from 22 additional African populations [17,24,25] (S1 Table and S1 Fig). This Pan-African dataset is comprised of populations from five broad geographical regions: West Africa, West-Central Africa, Great Lakes Africa, Horn of Africa, and Southern Africa (Fig 1A and S1 Table). Specifically, the West African region includes Gambian and Ghanaian tribes [17], and the West-Central African region includes Nigerian tribes (Yoruba and Igbo). The Great Lakes African region includes our Northern Ugandan (UNW and UNC) populations and also Southwest Ugandan, Kenyan and Tanzanian populations.

### Population structure and inferences of gene flow in West and West-Central Africa

Although our NCI Ghana set included individuals from approximately 35 tribes, ADMIXTURE results showed a homogeneous ancestry pattern (91% of the blue genomic ancestry Fig 1C and S6, S7, S10 and S12 Figs), similar to the Ga-Adangbe tribe, with the blue genomic ancestry being predominant in West-Central Africa (Fig 1C and S6–S8 Figs). We observed similar ancestry composition of Ghanaians and Nigerians, who both share predominant West-Central Africa ancestry (blue). In accordance with their more Western location, Ghanaians shared a minor proportion of West African ancestry (red genomic ancestry in Fig 1C) related to Gambian tribes, while the Yoruba and Igbo shared a minor ancestry proportion (purple, Fig 1C) related to Eastern Bantu populations from the Great Lakes Africa region. This pattern of ancestry in Yoruba and Igbo has been seen in recent studies [17, 26–28]. Our Ghanaian population showed negligible Eurasian admixture (S9 Fig) with mean Eurasian ancestry of 0.4%.

**Fig 2:**
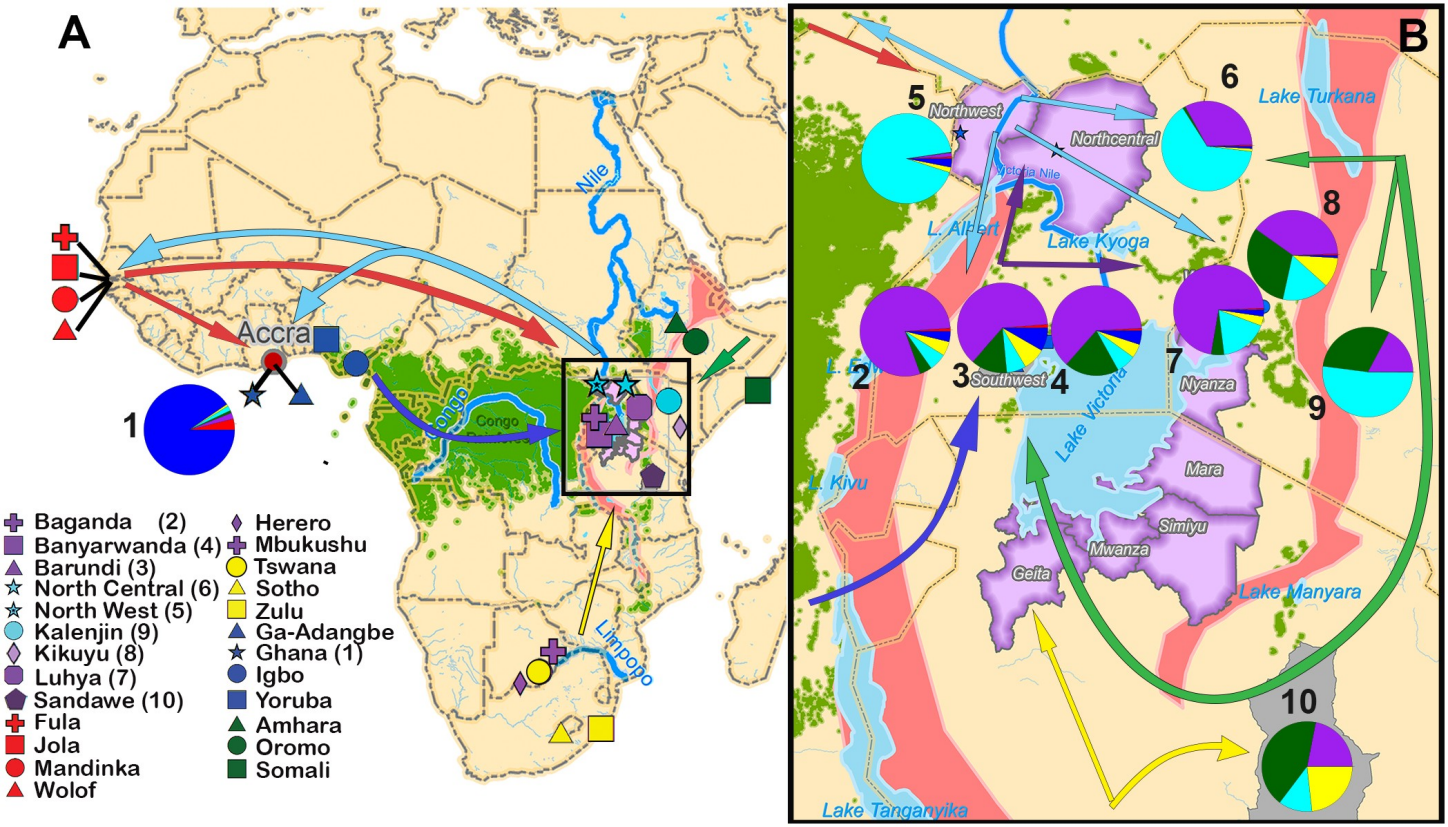
Populations movement routes in relation to major geographical barriers based on genetic inferences. (A) Map of Africa showing the geographical origin of the 22 previously reported Pan-African populations used herein, plus the three new populations from Ghana and the Uganda EMBLEM study (S1 Table). The arrows in the map indicate population gene flow based on significant f3 statistic tests (S2 Table). Colors of the arrows and the pie charts represent the ancestries inferred by ADMIXTURE (Fig 1C). Rather than shortest geographical distance, shapes of the arrows consider the major geographical barriers such as the Congo rainforest (light green), the East and West African Rift valley systems (pink), and the corresponding Rift Valley lakes and rivers (light blue). (B) The zoomed-out map shows the postulated dispersal routes in greater detail, as well as locations of the populations in the East African Rift Valley plateau sampled in previous studies (2 = Barundi, 3 = Banyarwanda, 4 = Baganda in Uganda; 7 = Luhya, 8 = Kalenjin, and 9 = Kikuyu in Kenya; and 10 = Sandawe in Tanzania), and the Uganda EMBLEM study populations [5 = Uganda North West (UNW), 6 = Uganda North Central (UNC)].

Consistent with ADMIXTURE inferences, both GLOBETROTTER analysis and the three-population test (ƒ_3_ statistic) inferred episodes of gene flow from Gambian tribes, and also from Nilotics, to Ghana and Nigeria that occurred during the last 4000 years (Fig 2, S13 Fig and S4 Table). The pattern of genetic structure in Ghanaians and Nigerians, and the inferred episodes of gene flow into West-Central Africa show that historical cultural exchanges between West and West-Central Africa [8, 9] and migrations across the Sahel (historical events i-iii of the Introduction) involving populations from East Africa have shaped the genetic composition of West-Central African populations.

### Population structure and inferences of gene flow in Uganda

The main feature of the genetic structure of Uganda shown by ADMIXTURE and PCA is the dichotomy between Northern Uganda populations, that show a predominantly Nilotic genomic ancestry (cyan ancestry in Figs 1 and 2 and S6–S9 Figs), and Southwest Uganda populations that have predominantly Eastern Bantu ancestry (purple ancestry, in Figs 1 and 2 and S6–S9 and S12 Figs). Within the predominantly Nilotic Northern Uganda populations, the UNW population is more homogeneous (93% Nilotic ancestry, Fig 1B and 1C and S7 Fig), while the UNC population is a mixture of Nilotic (64%) and Eastern Bantu genomic ancestry (Fig 1B and 1C and S7 and S12 Figs). Interestingly, Nilotic ancestry was detected in all Great Lakes African populations (Fig 1C and S7 Fig). In general, ADMIXTURE and PCA showed that the Great Lakes African region, which includes populations from Uganda, Kenya and Tanzania, was the most ancestry diverse region in sub-Saharan Africa (Fig 1B and 1C). Our Ugandan populations showed negligible Eurasian admixture (S9 Fig) with mean Eurasian ancestry of 0.02% in UNC and 0.015% in UNW.

GLOBETROTTER inferences suggest an episode of gene flow from West/West-Central Africa into UNW (849–936 years before present (YBP), 95% confidence interval, S13 Fig), although this was not confirmed by ƒ_3_ statistics (Fig 2 and S4 Table). In contrast to UNW, both ƒ_3_ (Fig 2 and S4 Table) and GLOBETROTTER (S13 Fig) consistently inferred several episodes of gene flow into the UNC and Southwest Uganda populations (Baganda, Barundi and Banyarwanda) from different sources: UNW (Nilotic), Southern Bantu, Horn of Africa (Cushitic), and also from West/West-Central African populations. GLOBETROTTER dates for these gene flow events (397–484 and 1499–2659 YBP, 95% confidence interval, S13 Fig) suggest two gene flow events that do not overlap with the inferred gene flow event into the UNW. We also inferred Nilotic-related (UNW and UNC) gene flow into Southwest Ugandan (Banyarwanda), Kenyan (Kikuyu and Kalenjin), Horn of Africa, West and West-Central African populations. Taken together, these results show that historical migrations (events iv-vi of the Introduction) of several human groups (Nilotic, Bantu and Cushitic) have shaped the current genetic composition in the Great Lakes region, and that Nilotic westward migration was accompanied by gene flow (historical event iii of the Introduction) (Fig 2 and S4 Table).

### Natural selection in two distinct eBL belt populations subject to major malaria burden

While our studied populations (Ghana and Northern Uganda) share a high incidence of malaria and eBL burden (S1 Table and S1 Fig) [22], our population structure analyses showed that they have distinct patterns of genetic ancestry (Fig 1). In order to understand if they share common signals of natural selection despite their differential genetic history, we searched for genomic signatures of natural selection in Ghana and Northern Uganda populations. The eBL cases were excluded from this analysis to eliminate confounding of natural selection results with disease associations. We applied the population branch statistic (PBS) approach [29] to each of these as a focal population, using the Southern Bantu Sotho and Zulu populations as a sister group and Europeans as the reference population (S15 Fig and see Methods). We used Southern Bantu populations as a sister group because, after the Bantu expansion in the last 2000 years, they have occupied an area outside the eBL belt, where the climate is drier and cooler, and thus not conducive for malaria transmission [30], also supported by a low reported frequency of malaria-associated variants [31]. We compared the PBS outlier values (99.9^th^ percentiles) against those generated by simulations of plausible neutral demographic models (Methods and S15–S17 Figs). In addition to the PBS statistic, we performed cross-population haplotype-based approach (xpEHH) to identify genomic regions under positive selection. We report as candidate selection regions those that showed extreme signal in both PBS and the xpEHH approach (above the 99.9th percentiles for PBS and >2 for xpEHH).

**Fig 3:**
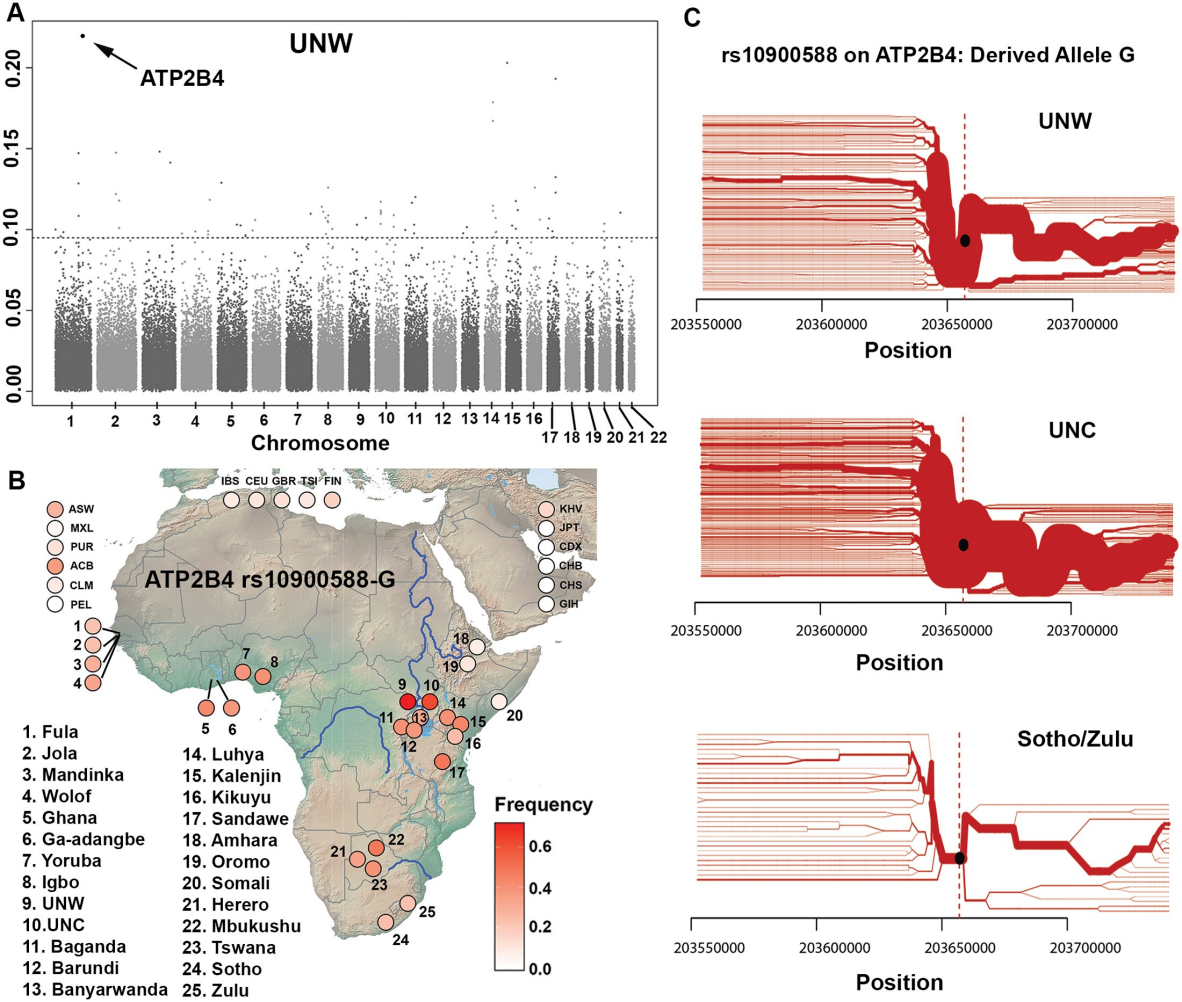
Malaria-driven natural selection analysis of rs10900588-G derived allele of gene *ATP2B4*. (A) Genome-wide population branch statistic (PBS). The mean PBS values (20 SNP windows) are represented by a Manhattan plot for Uganda North West (UNW). SNP rs10900588 on chromosome 1 showed the strongest signal of selection (See S15 Fig for LD graphs). (B) World-wide frequencies (red intensity) of the rs10900588-G derived allele, with map of the Pan-African plus UNW and UNC populations. (C) Haplotype bifurcation diagrams for the core haplotype at *ATP2B4* gene in Uganda North West (UNW), Uganda North Central (UNC), and Shoto/Zulu. ASW—Americans of African Ancestry in SW USA; MXL—Mexican Ancestry from Los Angeles USA; PUR—Puerto Ricans from Puerto Rico; ACB—African Caribbeans in Barbados; CLM—Colombians from Medellin, Colombia; PEL—Peruvians from Lima, Peru; KHV—Kinh in Ho Chi Minh City, Vietnam; JPT—Japanese in Tokyo, Japan; CDX—Chinese Dai in Xishuangbanna, China; CHB—Han Chinese in Beijing, China; CHS—Southern Han Chinese; GIH—Gujarati Indian from Houston, Texas.

We observed 14, 12 and 11 candidate genomic regions in the Ghanaian, UNW and UNC populations, respectively, (Fig 3, S16 Fig, and S5–S7 Tables), nominated by 32 index SNPs. While the Ghanaian sample yielded the largest number of candidate genomic regions, none of them were significant in the demographic model performed (S5 Table). Of 32 candidate genomic regions, seven are found within/adjacent the same gene and shared between two populations: *RARB* found in Ghana and in UNC (different index SNPs), and six genomic regions within/adjacent to *KLHL20*, *ATP2B4*, *NIT2*, *TENM3*, *GPHN* and *HERC2*, are found in both UNW and UNC (five of six regions share the same index SNP) (S5–S7 Tables).

The extreme PBS values came from the genomic region at the *ATP2B4* gene in UNW (p-value = 0.0011) and UNC (p-value = 0.0021) (Fig 3A), but not in Ghana or other eBL belt populations evaluated (S5 and S8 Tables). Analysis using the xpEHH statistic (based on the pattern of extended haplotype homozygosity (EHH) between populations) corroborates the PBS signal in *ATP2B4* gene for both UNC and UNW (Fig 3 and S6–S8 Tables). *ATP2B4* encodes the plasma membrane Ca^2+^-ATPase type 4 protein (PMCA4), the main calcium pump of the human erythrocyte [32]. Six SNPs within the genomic region in the *ATP2B4* gene (rs11240734-C, rs1541252-T, rs1419114-A, rs10900588-G, rs3851298-T, rs2228445-T) were detected as PBS outliers in both UNW and UNC. These six SNPs are located within two adjacent linkage disequilibrium (LD, r^2^ = 0.82) blocks of 6 and 12 Kb (S18 Fig). The intronic SNP rs10900588-G derived allele exhibited the highest PBS values in both Northern Uganda populations (Fig 3A and 3B). This SNP is within a core haplotype observed with high frequency in both Northern Uganda populations (UNW and UNC) and much lower frequency in the South Bantu Sotho and Zulu populations (Fig 3C and S18 Fig). Consistently, the highest frequencies in Africa of the rs10900588-G were observed in UNW (0.72), followed by UNC (0.63), and the lowest frequencies in the Horn of Africa (0.064–0.096), followed by Fula (0.22), Zulu (0.23) and Sotho (0.24) (Fig 3C).

The other five shared signals of candidate selection in Northern Ugandans (UNW and UNC) for the following genes: *KLHL20* (p-values 0.0021 UNW, and 0.0043 UNC), *NIT2* (p-values 0.0027 UNW and 0.0035 UNC), *TENM3* (p-values 0.0022 UNW and 0.0041 UNC), *GPHN* (p-values 0.0036 UNW and 0.0018 UNC) and *HERC2* (p-values 0.0016 UNW and 0.0031 UNC). None of these genes have clear relationship with malaria pressure and are likely related to other selective pressures in Northern Uganda, which are not explored in the current study.

## Discussion

Our study highlighted how the combined effects of demographic history and likely malaria-driven natural selection have shaped the genetic structure of populations in the eBL belt. We found evidence of gene flow events across the eBL belt in the last 3000 years, possibly related to regional migrations in Western Africa and major migrations involving Nilotic, Cushitic, and Bantu groups. Importantly, we identified for the first time in Africa a Northern Uganda-specific strong signal of malaria-driven selection in the *ATP2B4* gene.

### Migrations in Africa and the genetic structure of eBL belt populations

Our results showed that historical migrations (denoted as i-vi in the Introduction) have left signals in the genome of eBL belt populations. The historical interactions of diverse linguistic groups (pastoral Nilotic, Cushitic and farming Bantu) along lush migratory corridors in the Lake Victoria basin plateau [33] is reflected in the current genomic composition of Uganda, Kenya and Tanzania populations (Figs 1 and 2 and S13 Fig). In the context of the six historical events highlighted in the Introduction (i-vi), the observed pattern of genetic structure is consistent with Nilotic dispersion southward into the Great Lakes region (event v, Figs 1C and 2, and S7 Fig) and westward across the Sahel region (event iii), which may have led to historical contacts with West African populations [11,26,34,35]. Our results showed that Nilotic influence extends into the Great Lakes Africa region, and also to the Western African region, likely in the last 2000 years, as suggested by our GLOBETROTTER inferred dates (Fig 2,S7 and S13 Figs and S4 Table). The dichotomous pattern of ancestry between Northern Uganda (predominantly Nilotic) and Southern Uganda (predominantly Bantu) probably reflects the influence of the Nilotic migration into Northern Uganda, in contrast with the Bantu migration into Southern Uganda.

Our three dozen Ghanaian tribes showed high genetic homogeneity, but also evidence of gene flow from Gambian tribes in West Africa. Historically, the West and West-Central African regions have experienced extensive interactions between local kingdoms and tribes in the last 2000 years [8,9]. For Ghanaians, these interactions led some tribes to change their language due to social or economic motivation [7]. Local historical interactions such as these could explain the observed homogeneous genetic ancestry in Ghanaians. We inferred one gene flow event from Gambian tribes into Ghana and Yoruba about 1337–3022 YBP (S13 Fig). These inferred episodes of gene flow may be the signature of Mande migration into Ghana as part of trading networks [36], as well as of interactions of ancient populations along salt, gold, and slave trade routes [7,13].

### Natural selection driven by malaria in the eBL belt

To search for natural selection driven by malaria in the eBL belt, we used eBL burden as an indicator of populations exposed to high sustained *falciparum* malaria transmission in the eBL belt (Fig 1, S1 Fig and S1 Table). By comparing the populations with the highest malaria pressures versus those with no malaria, we identified for the first time a candidate region for malaria-driven selection in the *ATP2B4* gene in African, specifically Northern Ugandan populations (Fig 3 and S6–S8 Tables). *ATP2B4* is ubiquitously expressed in human tissues, and encodes the plasma membrane Ca^2+^-ATPase type 4 protein (PMCA4) [37], which is the most commonly expressed Ca^2+^ transporter in human erythrocytes [32]. We note that seven *ATP2B4* intronic SNPs (not present in our data) have been reported to be associated with multiple blood cell-related traits in African American, East Asian, European and Hispanic populations: mean corpuscular volume and hemoglobin concentration [38–41], lymphocyte counts, and red cell distribution width [38]. Furthermore, five of these seven *ATP2B4* SNPs (minor frequency alleles: rs10900585-G, rs2365860-C, rs10900589-A, rs2365858-G and rs4951074-A) were associated with resistance against severe *falciparum* malaria in Western African populations in Ghana and Gambia [42], and rs10900585 has been associated with reduced malarial placental infection and related maternal anemia in Ghana [43]. In an analysis of 11,890 cases of severe falciparum malaria and 17,441 controls from Africa, Asia and Oceania of 55 previously identified SNPs, rs10900585 was significantly associated with severe malaria over all African sites combined, and in the Ghanaian and Gambian samples [44]. Importantly, the protective minor alleles of these five SNPs above (not present in our data) are highly linked (mean *r*^2^ = 0.94) with the minor allele (as defined in non-Nilotic populations) of our strongest signal of selection (*ATP2B4* rs10900588-G) in the Luhya population (LWK) from the 1000 Genomes Project.

While polymorphisms in the *ATP2B4* gene were described as protective against severe malaria in Ghana and Gambia [42], the outlier approach used in the present study did not identify *ATP2B4* as a candidate selection gene in Ghanaians and Nigerians (S8 Table). This result is in accordance with the absence of natural selection signals in the *ATP2B4* gene reported for previous studies using samples from Western Africa [17, 45–49]. The lack of concordance between association studies and natural selection analysis can be explained by the fact that the frequency of the protective haplotype observed in Ghanaians is sufficient to identify significant disease association (a 6% difference between cases and controls across the protective haplotype) [42], but not sufficient to identify significant positive selection signal (an average 14% difference between West Central African and South African populations, compared to an average 45% difference between Northern Ugandan and South African populations, at rs10900588). In addition, when analyzing the *ATP2B4* association studies with cerebral malaria and severe malaria anemia in African, Asian and Oceanian populations, the Malaria Genomic Epidemiology Network [44] noted that the effect of the *ATP2B4* ancestral allele rs10900585-G on malaria might be heterogeneous across phenotypes and/or populations. The heterogeneity of effects may indicate presence of biological variation due to epistasis, gene-environment interactions, or that the analyzed SNP is in LD with an unknown causal allele associated with resistance to malaria. As LD patterns vary among populations, replication of the association would only be feasible if the causal SNP were genotyped.

The highest worldwide frequency of rs10900588-G allele and its related core haplotype observed in Northern Uganda populations (UNW and UNC, Fig 3 and S18 Fig) suggests a Northern Uganda- or Nilotic-specific selection in the *ATP2B4* gene, although the reasons for specificity are currently unclear to us. Consistent with this, our natural selection analyses using neighboring populations in Southern Uganda and Kenya did not identify signal of selection in the *ATP2B4* gene (S8 Table). The most likely explanation for this Northern Uganda-specific selection is that this region has historically experienced one of the highest levels of malaria infection worldwide (400–1,500 infectious mosquito bites per capita per year) [21]. A previous report has identified a signal of malaria-driven natural selection, at rs10900585 in the *ATP2B4* gene, by estimating the population-scaled selection coefficient in a time series of allele frequencies [50] in 92 ancient European samples from the Bronze Age (5000 bp) to the Post-Roman era [51], suggesting an ancient role of *ATP2B4* in malaria-driven selection.

The biological relationship between *ATP2B4* and malaria resistance is mediated by polymorphisms in *ATP2B4* changing PMCA4 structure or expression, which leads to a homeostatic disruption of intra-erythrocytic Ca^2+^ levels that are critical to the development of the *Plasmodium* parasite [42]. In an expression quantitative trait locus (eQTL) meta-analysis of whole blood gene expression [52], the allele rs10900588-G and linked SNPs were described as significant *cis*-eQTLs of *ATP2B4* (rs10900588-G with Z = -7.30, p-value = 2.91E-13, FDR = 0.00), i.e., the minor allele rs10900588-G is associated with significantly reduced *ATP2B4* expression. Recently, in a search for eQTLs enriched in human erythroblasts, Lessard *et al*. identified an erythroid-specific enhancer region just proximal to exon 2/alternate exon 1 of *ATP2B4* [53]. Lessard *et al*. demonstrated functional effects of the enhancer region through genome editing and *in vitro* cell culture, suggesting a Ca^2+^ homoeostasis defect as one possible pathway for the *ATP2B4* associations with malaria. The core haplotype we defined in the Northern Ugandan population extends from just proximal to exon 2/alternative exon 1 into intron 2/alternative exon 1. This haplotype overlaps with a minor *ATP2B4* haplotype in a European population (defined by the minor alleles in non-Nilotic populations of rs1541252, rs1541253, rs377342347, rs1419114, rs2228445, with mean *r*^2^ = 0.96 with rs10900588 in the LWK population) that results in reduced erythrocyte PMC4A expression and reduced Ca^2+^ export [54]. Both Lessard *et al*. and Zámbó *et al*. have suggested mechanisms by which reduced Ca^2+^ export may be related to reductions in malaria risk: Lessard *et al*. suggests erythrocyte dehydration as a resistance factor, while Zámbó *et al*. suggests that reduced Ca^2+^ export into the invaginated extracellular membrane reduces Ca^2+^ concentration, which is required for *Pfa* maturation. Supporting the suggested mechanism, the most recent report [55] showed a significant association between low *falciparum* malaria parasitemia and the homozygous genotype for the *ATP2B4* rs1541255-G allele (not present in our data). Importantly, this allele is in perfect LD (R^2^ and D’ = 1) with our most important *ATP2B4* signal (rs10900588-G) in Kenya.

There are extensive reports in the literature regarding selection pressure driven by malaria in the *HBB*, *ABO*, *DARC* and *G6PD* genes [44, 56]. It should be noted that, in the present study, the tests used for the detection of positive selection are based on assumptions such as high differentiation between populations (PBS) and hard selective sweeps (xpEHH). Therefore, it is important to emphasize that this is not the case for *HBB* and *ABO*, that are evolving under a balancing selection regime [56], nor is this the case for *DARC*, that despite being under positive selection, is almost fixed and with low differentiation among African populations [57]. Also, as we did not examine the X chromosome, *G6PD*, found on the X chromosome, was not investigated in the present study.

Although malaria is the presumed major driver of natural selection in the eBL belt populations (S1 Table), we understand that other selection pressures, which were not investigated in our study, might be acting on our study populations. For example, we found significant signal of selection in Northern Ugandans for the *OCA2/HERC2* and *NIT2* genes (Fig 3, and S6 and S7 Tables). The first is significantly associated with skin, eyes and hair pigmentation [18] and the latter is a potential tumor suppressor [58].

## Conclusions

After characterizing the genetic structure of the Ghanaian and Ugandan populations in the eBL belt, we showed that (i) historical interaction between West and West-Central Africa involved episodes of gene flow from West to West-Central Africa; (ii) the documented cultural interaction between the local kingdoms of West-Central Africa, specifically in Ghana, were accompanied by an homogenization of the gene pool of these populations, independently of their linguistic diversity; (iii) the pattern of genetic diversity of the eBL belt populations show the signature of migrations across the Sahel that include Nilotic expansion into West Africa; (iv) the genetic composition of Great Lakes African populations is the result of the interactions between Nilotics, Cushitics and Bantu groups in the last 3000 years; and, (v) the *ATP2B4* gene, which was previously associated with erythroid-related traits and malaria susceptibility, shows the signature of malaria-driven natural selection specific to Northern Uganda (UNW and UNC). These results provide important baseline genomic data to facilitate disease association studies, including of eBL, in eBL belt populations.

## Methods

### Ethics statement

Ethical approval for EMBLEM was obtained from the Uganda Virus Research Institute Research and Ethics Committee, the Uganda National Council for Science and Technology (H816), and the NCI Special Studies Institutional Review Boards (10-C-N133). The Ghana Prostate Health Survey was approved by the Noguchi Memorial Institute for Medical Research Institutional Review Board (001/01-02) and by the NCI SSIRB (02CN240). Participants in both the EMBLEM and Ghana Prostate Healthy Study gave informed written consent.

### Ugandan and Ghanaian samples, genotyping and data curation

The NCI Ghana set included random samples of 964 healthy men from approximately 35 tribes (S2 Table) aged 50–74 years old enrolled for prostate cancer screening into the Prostate Healthy Survey [59]. The Ugandan samples were from 758 children aged 0–15 years old (including 197 eBL cases and 561 controls) from 13 tribes enrolled in the **E**pide**m**iology of **B**urkitt **L**ymphoma in **E**ast-African Children and **M**inors (EMBLEM) study in two regions of Northern Uganda (Uganda North West [UNW] and Uganda North Central [UNC]). The healthy children were enrolled from 100 randomly selected villages in these regions (S3 Table) [60]. The samples were genotyped using the Illumina Infinium HumanOmni5-4v1 genotyping array in the Cancer Genomics Research Laboratory (CGR) at the National Cancer Institute (NCI); quality control was performed using PLINK 1.07 software [61] and in-house scripts [62].

### Relatedness

We calculated the inbreeding (F) and the kinship coefficients (Φ_ij_) using the PLINK 1.07 software [61] (S2 and S3 Figs). Following Kehdy et al. [24] a Φ_ij_ threshold ≥ 0.1 was used to create family networks (S2 and S3 Figs) and we excluded interactively individuals with the highest number of relatives, which allow us to reduce family structure, minimizing sample loss. Following this procedure, we created “unrelated” NCI Ghana and Ugandan datasets (S1 Table).

### Merging genotyping data

We merged the NCI datasets (1,513 individuals with >48 tribal affiliations) with public African genome-wide datasets, creating a Pan-African dataset (PA dataset) of 1,287,642 SNPs for 3102 individuals, from 9 countries, and 11 ethnolinguistic groups in Sub-Saharan Africa (S1, S2 and S3 Tables). We also merged the PA dataset with all 1000 Genomes Project Phase 3 populations [24] creating the PA1KGP dataset, to test the extent of Eurasian admixture in the NCI datasets.

### Population structure and demographic history

Since ADMIXTURE software [63] assumes independence among genetic markers, we used PLINK 1.07 to prune the SNPs in high linkage disequilibrium (LD) using a pairwise linkage disequilibrium maximum threshold of 0.4, a window size of 50, and a shift step of 10, creating the PA non-LD dataset with 727,834 SNPs. Then, we used the PA non-LD dataset to perform ADMIXTURE [63] and Principal Components Analysis (PCA) [64]. To verify possible sample size effects on ADMIXTURE and PCA analysis [65], we resampled the PA non-LD dataset to reach similar number of individuals for each studied population (S8 and S11 Figs).

We phased the PA dataset using SHAPEIT [66]. Using the phased dataset, we performed fineSTRUCTURE [67] analysis (10 million iterations of Markov chain Monte Carlo) to determine the genetically homogeneous groups and GLOBETROTTER [68] to infer historical admixture events.

We also estimated the *ƒ*3 statistic to infer events of gene flow and their possible directions, as implemented in the software ADMIXTOOLS [69], for all possible combinations of three populations using the PA dataset. All *ƒ*3 statistics with Z-score ≤ -3 were considered as highly significant evidence of gene flow. For the *ƒ*3 statistic and GLOBETROTTER analysis of historical gene flow events, we described contributing ethnic groups or populations with the suffix “-like”, representing present day surrogates of the real sources [67]. Masterscripts used for data curation and population structure analyses are available at the EPIGEN-Scientific Workflow (http://ldgh.com.br/scientificworkflow/, [62]).

### Natural selection

To search for genomic footprints of selection in Ghana and Uganda, we explored allele frequency differentiation using Population Branch Statistic (PBS) using all the data, i.e., without LD pruning as done during the PCA and ADMIXTURE analysis [29], but excluding the eBL cases in Northern Uganda. PBS estimates were performed using NCI Ghanaians and Northern Ugandan controls as study populations, the Southern Bantu populations (Sotho and Zulu) from the African Genome Variation Project [17] as a sister group, and the Europeans (CEU+TSI+FIN+GBR+IBS) from 1000 Genomes project [24] as reference population.

In addition to PBS, we performed Extended Haplotype Homozygosity (EHH) [70] analysis (SI) using the Cross-population Extended Haplotype Homozygosity (xpEHH) [71] in R package rehh v.2.0.2 [72]. To minimize spurious results of individual SNPs [73], all the selection analyses were performed on windows of 20 SNPs overlapping by 5 SNPs. For the density of SNPs used in the present study (~1,000,000), the average window size of 20 SNPs corresponds to an average ~ 50 Kb. We used ANNOVAR [74] to annotate SNPs found in candidate regions under selection. To consider a candidate region to be under selection, we adopted a conservative approach of filtering those regions that showed extreme signals in both PBS and xpEHH methods (S5–S7 Tables). For the intergenic natural selection signal, we represented the genetic distances from the closest genes (S5 and S6 Tables).

### Simulations of the neutral coalescent model

Simulations were carried out using the demographic model [76] (S15 Fig), based on estimated divergence (thousands of years ago, kya) and effective population size (Ne) of African populations performed in Mallick *et al*. [75]. We used the Dinka population as a proxy for UNC and UNW, and the Luhya population as a proxy for Southern Bantu, with inferred divergence range of 9 and 25 kya (Mallick *et al*. high and low divergence inference), and current Dinka and Luhya Ne of 3x10^4^ and 3x10^4^, respectively [75]. We used the Yoruba population as a proxy of the Ghanaian population, and the estimated divergence from the Luhya of 5 and 10 kya and current Yoruba Ne of 7x10^4^. We also used the French population as a European proxy, 40 to 60 kya for an inferred divergence time and 3x10^4^ for current Ne. Considering that the study populations were involved in gene flow events, we introduced migration parameters between study populations and Southern Bantu considering the ancestry proportions inferred by ADMIXTURE (Fig 1C), as 4Nem_ij_, where 4Ne is the population effective size and m_ij_ the fraction of population *i* that is made up of migrants from population *j* (for more details see S15 Fig).

Additional Methods are presented in Supporting Information (S1 Text).

## Supporting information

S1 Text Additional information and methods. (DOCX)

S1 Fig All studied populations in relation to the endemic Burkitt lymphoma (eBL) belt as represented in the original paper by Haddow et al [31]. The eBL belt is shown in red shade and the incidence of eBL is denoted by the red color intensity.
(TIF)

S2 Fig Kinship and inbreeding in the Uganda (EMBLEM) and Ghana datasets. (A) Kinship coefficients (Φ_ij_) estimates by the probabilities of IBD = 0 estimates for all pairs of individuals. The colored dots are the theoretical relatedness degree probabilities of Φij and IBD = 0. (B) The distribution of individual inbreeding coefficients estimated for all individuals.
(TIF)

S3 Fig Inbreeding in the EMBLEM Uganda sample separated by Burkitt lymphoma (Cases), pilot population controls (PPCs), matched population controls (MPCs) and health-center II controls (HCII). (TIF)

S4 Fig Representation of the virtual families (genomic inferences) in the Uganda dataset by complex networks. We represented in the same image the individual inbreeding coefficient and the pairwise kinship coefficient (Φ_ij_) that represents the relatedness among the individuals. In this network, the nodes are the individuals and the edges are kinship relationships between individuals. Here, we linked only pairs of individuals with Φ_ij_ ≥ 0.06 (A) or ≥ 0.1 (B), which means we consider as related only individuals with relatedness ≥ third or second degree, respectively. The size of nodes is proportional to the absolute value of individual inbreeding and the shape of the node serves to signal whether inbreeding is positive (square) or negative (circle). The colors of the nodes represent the Uganda individual’s tribe (S1A Table). We represented only the samples with proportion of identity by descent (Plink PI_HAT) > 0.05.
(TIF)

S5 Fig Representation of the virtual families (genomic inferences) of Ghana dataset by complex networks with Φ_ij_ ≥ 0.06 (A) or ≥ 0.1 (B). In each network, the nodes are the individuals and the edges are kinship relationships between individuals. We represented only the samples with proportion of identity by descent (Plink PI_HAT) > 0.05.
(TIF)

S6 Fig ADMIXTURE barplot representation of the individual ancestry proportions of the Pan-African populations. (Top) The proportions of individual ancestry values were calculated using ADMIXTURE unsupervised mode with the number of ancestral K = 2 to K = 15. (Bottom) ADMIXTURE cross-validation errors as a function of K.
(TIF)

S7 Fig Mean ancestry composition inferred by ADMIXTURE for Ghanaian and Ugandan populations in relation to 19 Pan-African populations. The populations are: Ghana; Uganda North West; Uganda North Central; and three populations from Uganda South West (Baganda, Barundi, and Banyarwanda). The Pan-African populations are from West Africa (Fula, Mandika, Wolof, and Jola), West Central Africa (Ga-Adangbe, Yoruba, and Igbo), Southern Africa (Mbukushu, Herero, Tswana, Zulu, and Sotho), Horn of Africa (Amhara, Oromo, and Somali) and Great Lakes Africa (Luhya, Kalenjin, and Kikuyu).
(TIF)

S8 Fig ADMIXTURE barplot representation of the individual ancestry proportions of the resampled PA non-LD dataset with similar number of individuals for each studied population. (TIF)

S9 Fig ADMIXTURE barplot representation of the individual ancestry proportion of the Pan-African populations combined with 1000 Genomes Phase 3 populations. We represented the unsupervised ADMIXTURE analysis with the number of ancestral clusters K = 10. This K captured the six African clusters represented in Fig 1C, and East Asian (pink), and South Asian (light green) Asian, European (black) and Native American (orange) ancestral clusters. ASW—Americans of African Ancestry in SW USA; MXL—Mexican Ancestry from Los Angeles USA; PUR—Puerto Ricans from Puerto Rico; ACB—African Caribbeans in Barbados; CLM—Colombians from Medellin, Colombia; PEL—Peruvians from Lima, Peru; KHV—Kinh in Ho Chi Minh City, Vietnam; JPT—Japanese in Tokyo, Japan; CDX—Chinese Dai in Xishuangbanna, China; CHB—Han Chinese in Beijing, China; CHS—Southern Han Chinese; GIH—Gujarati Indian from Houston, Texas.
(TIF)

S10 Fig Principal component analysis (PCA) of the Pan-African populations. We compared the following PC combinations: PC1 vs PC2, PC3 vs PC4, PC1 vs PC3 and PC2 vs PC4.
(TIF)

S11 Fig Principal component analysis of the resampled PA non-LD dataset with similar number of individuals for each studied population. We compared the following PC combinations: PC1 vs PC2, PC3 vs PC4, PC1 vs PC3 and PC2 vs PC4.
(TIF)

S12 Fig Haplotype clustering analysis. (A) fineSTRUCTURE tree and (B) heatmap of the length of the chunks shared by individuals. Each row of the heatmap represent a copyvector of a recipient individual and each column represent the proportions of haplotypes that a donor shares with a recipient. Dark regions of the heatmap represent the long haplotype segments shared between individuals. Dark regions outside on the diagonal indicate more recent gene flow events. We highlight the inferred clusters using the colors of the ADMIXTURE ancestries.
(TIF)

S13 Fig Admixture events inferred by GLOBETROTTER for West Central Africans (Ghana, Yoruba and Igbo) and Ugandan populations. Ancestry profiles and admixture dynamics were inferred using non-local donors. Donor populations were selected based on fineSTRUCTURE results. In the mixture model and event sources, the bars show the contribution of each African population to the recipient populations. The plot on the left represents the most likely estimated admixture dates inferred by GLOBETROTTER. The plot shows two admixture events, except in the West Africans and North West Uganda where only one admixture event was found. Inferred date(s) and 95% CIs are represented by the dots and horizontal lines in the graph. Bars corresponding to the event sources represent the inferred admixing sources for each estimated admixture event and the proportion of contribution of the African donor populations.
(TIF)

S14 Fig Coancestry curves of GLOBETROTTER inferences for Ugandan and Ghanaian populations. These curves are informative for date estimation and the genetic composition of the sources of the admixture event. Each curve describes the probability to find two chunks of two donor populations along the genome of the target population. Curves with decreasing probability indicate that the two donor populations describe the genetic composition of one source population. Increasing probability indicates that the two donors could describe different sources. One date admixture (North West and Ghana) is characterized by a uniform curve that decreases or increases its probability. On the other hand, multiple date admixture (North Central) is characterized by a curve that changes its behavior, for example, from increasing (indicating different donor for the earlier event) to decreasing (both donor populations describe one source for the recent event).
(TIF)

S15 Fig Neutral coalescent demographic model used in the PBS analysis. Ne = effective population size, kya = thousand years ago and *m* = migration rate. We used the migration rates following the current ancestry profile estimated by ADMIXTURE, as 4Nem_ij_, where 4Ne is the population effective size and m_ij_ the fraction of population *i* that is made up of migrants from population *j*. The simulated genotypes were obtained by the ms program [76] with the following command line: ms NTotalPop 10000 –s 1 –t 0.01 –I 3 NPop1 NPop2 NPop3 –eg 0.002 1 gPop1 at time1 –em 0.002 1 3 NMigrants_*ij*_*−*em 0.002 3 1 NMigrants_*ji*_ -ej 0.025 3 1 –en 0.06 1 gPop1 at time2 –en 0.06 1 gPop2 at time2 –ej 0.06 2 1 –en 0.1 1 gPop1 at time3.
(TIF)

S16 Fig The mean PBS values (by 20 SNP windows) represented by the Manhattan plots for (A) GHANA, (B) Uganda North West (UNW) and (C) Uganda North Central (UNC). The dotted line demarcates the 99.9th percentile. The red dots represent genes that also have a selection signal by the xpEHH test (>2).
(TIF)

S17 Fig *ATP2B4* PBS value observed against neutral distribution. PBS neutral values were generated by 10,000 simulations of plausible neutral demographic models (S15 Fig) for UNW and UNC populations respectively.
(TIF)

S18 Fig Haploview LD table and core haplotype (rs10900588) of the ATP2B4 genomic region with the extreme PBS values (Fig 3A). We presented the LD table of the Uganda North West (UNW) population that showed the highest signal of selection.
(TIF)

S1 Table Pan-African population samples used in the study and the malaria risk estimated by metrics of malaria prevalence and number of months in year with intense malaria transmission and eBL burden information for each population. (XLSX)

S2 Table Self-reported tribes of participants in the NCI Ghana Prostate Health Survey. (XLSX)

S3 Table Self-reported tribes of participants in the EMBLEM study in Northern Uganda. (XLSX)

S4 Table Admixture signal in Ghanaian, Ugandan, Kenyan and Horn of Africa populations using the three-population test (*ƒ3* statistic). The three-population test (*ƒ3*) statistic evaluates if the allele frequencies of a target population are intermediate of two sources which is interpreted as a result of admixture. Evidence of admixture is represented by negative *ƒ3* values; significant evidence of admixture is inferred with a Z-score <-3. All populations evaluated in the global ADMIXTURE analysis were included. In addition, several populations were combined and evaluated as sources or target to assess broader groups (i.e., West_Central_Ghana, Banyarwand_Barundi). Only combinations that resulted in Z score ≤-3 are tabulated.
(XLSX)

S5 Table Gene candidates for natural selection in the Ghana population based on the outliers PBS (99.9th percentile and p-value<0.05) and xpEHH (>2) tests. The SNP with the highest PBS value for each candidate gene is tabulated.
(XLSX)

S6 Table Gene candidates for natural selection in the Uganda North West (UNW) population based on the outlier PBS (99.9th percentile and p-value<0.05) and xpEHH (>2) tests. The SNP with the highest PBS value for each candidate gene is tabulated.
(XLSX)

S7 Table Gene candidates for natural selection in the Uganda North Central (UNC) population based on the outlier PBS (99.9th percentile and p-value<0.05) and xpEHH (>2) tests. The SNP with the highest PBS value for each candidate gene is tabulated.
(XLSX)

S8 Table PBS and xpEHH test values for the *ATP2B4* gene in Ghana, Nigeria, Uganda and Kenya. (XLSX)
