# Supplementary material for: Genetic signatures of gene flow and malaria-driven natural selection in sub-Saharan populations of the "endemic Burkitt Lymphoma belt"
Source: PLoS Genet. 2019 Mar 8;15(3):e1008027. doi: 10.1371/journal.pgen.1008027 (PMC6426263; doi:10.1371/journal.pgen.1008027)
Supplement: S1 Text — (DOCX) [file pgen.1008027.s001.docx]

Genetic signatures of gene flow and malaria-driven natural selection in Sub-Saharan populations of the “endemic Burkitt Lymphoma Belt”

**Supporting Information (SI)**

**EMBLEM Uganda and Ghana Samples**

The EMBLEM Uganda samples included 197 eBL cases enrolled from the general population served by St. Mary's Hospital Lacor, Gulu, and Kuluva Hospital, Arua, in Uganda North Central (UNC) and Uganda North West (UNW), respectively, and 561 healthy children aged 0-15 years old enrolled from 100 random villages in the same regions as eBL cases [1]. Basic demographics, clinical history and a venous blood sample in EDTA tubes for genetic testing were obtained from all participants. The study was approved by the Uganda Virus Research Institute Research and Ethics Committee, the Uganda National Council of Science and Technology (protocol #HS816), and the National Cancer Institute Special Studies Institutional Review Boards (NIH Protocol #10CN133) and all subjects provided written informed consent or assent (for children aged 8 years or more).

The National Cancer Institute Ghana (Ghana) samples included 964 randomly sampled men aged 50-74 years old who participated in a Prostate Health Survey in Accra, Ghana [2]. Basic demographic data and a venous blood sample in ACD tubes for genetic testing were obtained from participants. The study was approved by the Noguchi Memorial Institute for Medical Research Institutional Review Board, and the National Cancer Institute Special Studies Institutional Review Boards (NIH Protocol #02CN240-A) and all subjects provided written informed consent.

**Genotyping and Quality control of the Genome-wide Data**

For the NCI studies, DNA extraction and high-throughput, genome-wide SNP genotyping, using Infinium HumanOmni5-4v1 (Illumina Inc. San Diego, CA), was performed at the Cancer Genomics Research Laboratory (CGR). Genotyping was performed per the manufacturer’s guidelines using the Infinium automated protocol.

For quality control, 6% of the samples were duplicated and we obtained an overall genotype concordance of > 99.9% using GenomeStudio software. Then, the genotype calls of the Uganda and Ghana datasets were exported in the PLINK [3] format using the Illumina GenomeStudio software. Quality control was performed based on concordance of technical duplicates. After that, we separated the 22 autosomes from the Mitochondrial, X and Y chromosomes. Then, we used the autosomal data to remove duplicate SNPs, and replace the Illumina’s “kgp” SNP identifiers to dbSNP’s “rs” SNP code using as reference the correspondence kgp/rs table provided by Illumina. Next, we used the PLINK software [3] to exclude the individuals with genotyping error rate per individual > 0.1 and also the SNPs with genotyping error rate per SNP > 0.1. This QC filters resulted in the exclusion of 128,493 SNPs, but did not exclude any individual from the EMBLEM dataset. The specific Ghana QC procedures applied to the Ghana dataset have previously been described in Cook et al [4]. After these quality control steps, we created an EMBLEM work dataset (EW dataset) comprised of ~4.3 million autosomal SNPs on 744 individuals and a Ghana work dataset (GW dataset) comprised of ~4.3 million autosomal SNPs on 964 individuals.

For data curation and population structure analyses we used the Masterscripts “Cleaning process”, “Merging genotype datasets”, “Quality Control -SDQC”, and “Smart Eigenstrat” and flowcharts “Ancestry”, “Cleaning process”, and “Merging Datasets” which are available at the EPIGEN-Scientific Workflow (http://ldgh.com.br/scientificworkflow/, [5]).

**Inbreeding and Relatedness**

To perform Identity by Descent (IBD) inferences for the work datasets, EW and GW, we pruned SNPs in linkage disequilibrium (r2 ≥ 0.1) using PLINK. For this pruning, we used the flag --indep-pairwise with three parameters 200 25 0.1. Thereafter, we inferred the inbreeding and the kinship coefficients (Φij) among all pairs of individuals using the IBD estimations implemented in PLINK [3]. The mean levels of inbreeding in the combined UNC and UNW populations, and the Ghanaian population, were 0.002 and 0.004, respectively (S2 Fig), corresponding to moderately high rates of consanguineous marriages (4-8%) [6]. We did not observe differences (Wilcoxon test) in the mean inbreeding coefficients between Northern Ugandan (UNW and UNC) eBL cases and controls (S3 Fig), consistent with the low familial risk of eBL [7]. The pairwise kinship coefficient Φij was used to link related individuals and create family networks following Kehdy et al. [8]. Consistent with the study designs (EMBLEM Uganda and Ghana Samples section), we observed more genetically related individuals (first, second, and third degree) in the UNW and UNC than Ghanaian populations (S2 Fig). Most relatives self-identified to the same tribe, however, there were a few who reported belonging to different tribes (S4 Fig), underscoring limited value of self-reported tribe information as a surrogate of genetic relatedness. We used the method implemented in the software NAtoRA (Network Algorithm to Relatedness Analysis) to reduce family structure by excluding the minimal number of related individuals [8]. Specifically, we used the Φij threshold ≥ 0.1 to define the families, in which all second and first degree relatives are linked and then, we excluded interactively the individuals with more relatedness links (the most central individuals in the network) to reduce family structure of the EW and GW dataset creating an unrelated dataset U.The U dataset for EW included 568 individuals, while the unrelated GW dataset included 945 individuals.

**Merging datasets**

We merged the unrelated U datasets of EW and GW (1,513 individuals with >48 tribal affiliations) with public African genome-wide datasets from the African Genome Variation Project [9], and the Tishkoff laboratory [10] (S1 Table), creating a Pan-African dataset (PA dataset) of 1,287,642 SNPs for 3102 individuals, from 9 countries, and 11 ethnolinguistic groups in Sub-Saharan Africa (S1, S2 and S3 Tables). Also, we merged the PA dataset with all 1000 Genomes Project Phase 3 populations [11] creating the PA1KGP dataset, to reveal the level of Eurasian admixture in the NCI datasets.

**Population structure analyses**

We performed Principal Components Analysis (PCA) and ADMIXTURE. Both analyses assume independence among the genetic markers; for this reason, we removed SNPs in high LD (r² > 0.4) from the PA dataset using PLINK with the flag indep-pairwise 200 25 0.4 to generate the PA non-LD dataset consisting of 727,834 SNPs. Using this dataset, we ran the PCA using EIGENSTRAT [12] software and the genetic clustering analysis using ADMIXTURE [13] in the unsupervised mode for values from 2 to 10 and evaluated model accuracy using the cross-validation test.

We inferred the haplotypes from all African populations using the SHAPEIT software [14]. Because SHAPEIT uses LD information for the data phasing, we used the PA dataset (without LD pruning), to create the haplotype PA dataset. In order to infer fine-scale patterns of population structure and admixture events, we used fineSTRUCTURE/GLOBETROTTER methods, respectively [15,16]. Both methods used CHROMOPAINTER [15] outputs that take advantage of the LD and the haplotype inference. Considering that these methods are highly computationally demanding and that we observed a homogeneous ancestry of Ghanaians (Fig 1C), we randomly sampled 200 individuals of 945 of our GW dataset and kept all samples for other populations.

At first, we performed the CHROMOPAINTER inferences to detect patterns of shared haplotypes among all individuals by reconstructing the haplotype recipient with donated chunks (DNA segments) from other haplotypes (chromosome painting) [15]. First, we ran CHROMOPAINTER to estimate two scaling parameters, the ‘recombination scaling constant’ (n) and the mutation parameter (Θ), using four chromosomes (1, 8, 15 and 21), a subsample of the haplotype PA dataset (15 individuals per population), and 10 iterations of expectation-maximization algorithm (EM). After calculating these two parameters, we ran CHROMOPAINTER with these fixed parameters (n=488.54 and Θ=0.0007786) for all chromosomes to generate chunkcounts and chunklengths co-ancestry matrices. The chunkcounts and chunklengths co-ancestry matrices explain the number and length of DNA segments that each individual share with each other individual of the dataset, respectively. These segments could be interpreted as identical by descent. Then, to detect fine-scale population structure, we used the chunkcounts co-ancestry matrix to perform the model-based Bayesian clustering implemented in the fineSTRUCTURE software [15]. We ran fineSTRUCTURE using 10,000,000 of burn in steps, 10,000,000 of Monte Carlo Markov Chain (MCMC) iterations and 100,000 resamplings. After the MCMC calculations, fineSTRUCTURE considered the configuration of clusters with the highest probability and performed 100,000 additional hill-climbing steps to construct a final tree of clusters. The organization of clusters of fineSTRUCTURE tree agreed with the ADMIXTURE K=6, moreover it showed correspondence between the populations and clusters (samples from population X clustered with samples from the population).

**Shared Evolutionary History Analysis and Inference of gene flow events**

To infer the gene flow events for Northern Uganda and NCI Ghana populations, we employed two different approaches: GLOBETROTTER [16] and the three-population test (ƒ3 statistics) [17,18].

GLOBETROTTER infers the ancestry proportions (MIXTURE MODEL) and estimates the date and mode of gene flow. For these analyses, the inclusion of closely related populations can mask the inference of gene flow showing a genealogical relationship instead of real gene flow. Thus, we performed the chromosome painting for GLOBETROTTER analyses excluding local donors, for example, in the case of Ghanaians, we did not include other West-Central African donors (Yoruba and Igbo), and for Ugandan populations (UNW, UNC, Baganda, Barundi and Banyarwanda) we did not use Ugandan donors. We used the clusters inferred by fineSTRUCTURE as potential donors (S12A Fig). For these algorithms, it is common to use the terminology of donor and recipient and these terms seem to suggest a direction of gene flow (from donor to recipient); but, in fact they show the haplotype similarity that could be due to real gene flow or common ancestry.

To understand the admixture dynamics in West Africa and Great African Lakes, we ran GLOBETROTTER analysis for Ghana, West-Central African populations (Yoruba and Igbo) and all Ugandan populations (UNW, UNC, Baganda, Barundi and Banyarwanda). GLOBETROTTER uses as input the chunklengths co-ancestry matrix and painting samples outputs generated by CHROMOPAINTER. For West (Ghana) and West-Central African populations (Yoruba and Igbo), to generate the chunk-lengths coancestry matrix, we ran CHROMOPAINTER analysis excluding the all West (Ghana) and West Central African (Yoruba and Igbo) as donors and all other African populations (including Ugandan populations) as donors and recipients. For the painting samples, we ran CHROMOPAINTER twice for each population, a first run to estimate the ‘recombination scaling constant’ (n) and the mutation parameter (Θ) specific for each e population using 30 EM-iterations and a second run in which we set the particular population as recipient from all other African populations except Ghana, Yoruba and Igbo donors. For Ugandan populations, we carried out the same approach as for West and West-Central African populations, we generated the chunklengths co-ancestry matrix setting the Ugandan populations as recipient and all other populations (including West-Central African populations) as donors and recipients.

After the CHROMOPAINTER runs, we used the chunklengths co-ancestry matrix to run the MIXTURE MODEL approach implemented in GLOBETROTTER to understand the pattern of shared haplotypes of the study populations. This approach uses non-negative least squares to calculate the proportion of haplotypes that a target population shared (or copied) from a potential donor. To estimate the date and mode of the possible admixture events, we used GLOBETROTTER. This software is based on linkage disequilibrium decay and it has the power to detect the last event or the last two events of admixture over the last 400 generations. GLOBETROTTER can infer the following modes of admixture: one date, one date multiway, multiple date admixture, uncertain, and no admixture. For this purpose, we use the information from the chunklengths co-ancestry matrix and painting samples to generate co-ancestry curves that contain the information of the admixture sources. These curves represent the probability of finding two chunks from different populations along the genome providing information about the date and admixture mode. With this information, we can reconstruct the genetic profile of the sources that contributed to a target population. In order to improve the date of admixture, the co-ancestry curves were calculated in two ways, with and without considering the standardization of “NULL” individuals. This standardization tried to eliminate spurious LD patterns. The confidences intervals were inferred using the results of bootstrap resampling (100 samples) (Fig 2A and S13 Fig).

Another approach to infer admixture and possible directions of the gene flow is the *f3* statistic (Three population test) [17,18]. This test is based on allele frequency variances and evaluates whether the SNP allele frequencies of a target population is the mixture of two possible sources. Briefly, it compares the allele frequency variance between the target with possible sources and the allele frequency variance between the possible sources. If the test resulted in a positive value, this means that the differences between the target and the sources are almost the same or higher than the differences among the possible sources, which indicates no admixture occurring. On the other hand, if the differences between the target and the possible sources are minimal compare to the differences among the sources, this result in a negative value which indicates admixture.

The *f3* statistics is sensitive to genetic drift, which can cause highly differentiation between the target and the possible sources, therefore a positive value of *f3* does not necessarily mean no admixture. With the Pan-African dataset we ran all possible combinations of three populations involving Ugandan and Ghanaian populations using the software package ADMIXTOOLS [18]. This test uses a block jackknife procedure to account for linkage disequilibrium and obtain estimates of statistical significance. All *f*3 values with a Z-score < -3 were considered as highly significant evidence of admixture.

**REFERENCES**

1. Maziarz M, Kinyera T, Otim I, Kagwa P, Nabalende H, Legason ID, et al. Age and geographic patterns of Plasmodium falciparum malaria infection in a representative sample of children living in Burkitt lymphoma-endemic areas of northern Uganda. Malar J. 2017;16: 124.

2. Hsing AW, Yeboah E, Biritwum R, Tettey Y, De Marzo AM, Adjei A, et al. High prevalence of screen detected prostate cancer in West Africans: implications for racial disparity of prostate cancer. J Urol. 2014;192: 730–735.

3. Purcell S, Neale B, Todd-Brown K, Thomas L, Ferreira MAR, Bender D, et al. PLINK: a tool set for whole-genome association and population-based linkage analyses. Am J Hum Genet. 2007;81: 559–575.

4. Cook MB, Wang Z, Yeboah ED, Tettey Y, Biritwum RB, Adjei AA, et al. A genome-wide association study of prostate cancer in West African men. Hum Genet. 2014;133: 509–521.

5. Magalhães WCS, Araujo NM, Leal TP, Araujo GS, Viriato PJS, Kehdy FS, et al. EPIGEN-Brazil Initiative resources: a Latin American imputation panel and the Scientific Workflow. Genome Res. 2018; doi:10.1101/gr.225458.117

6. Bittles AH. Endogamy, consanguinity and community genetics. J Genet. 2002;81: 91–98.

7. Brubaker G, Levin AG, Steel CM, Creasey G, Cameron HM, Linsell CA, et al. Multiple cases of Burkitt’s lymphoma and other neoplasms in families in the North Mara District of Tanzania. Int J Cancer. 1980;26: 165–170.

8. Kehdy FSG, Gouveia MH, Machado M, Magalhães WCS, Horimoto AR, Horta BL, et al. Origin and dynamics of admixture in Brazilians and its effect on the pattern of deleterious mutations. Proc Natl Acad Sci U S A. 2015;112: 8696–8701.

9. Gurdasani D, Carstensen T, Tekola-Ayele F, Pagani L, Tachmazidou I, Hatzikotoulas K, et al. The African Genome Variation Project shapes medical genetics in Africa. Nature. 2015;517: 327–332.

10. Crawford NG, Kelly DE, Hansen MEB, Beltrame MH, Fan S, Bowman SL, et al. Loci associated with skin pigmentation identified in African populations. Science. 2017; Available: ttp://science.sciencemag.org/content/early/2017/10/11/science.aan8433.abstract

11. 1000 Genomes Project Consortium, Auton A, Brooks LD, Durbin RM, Garrison EP, Kang HM, et al. A global reference for human genetic variation. Nature. 2015;526: 68–74.

12. Price AL, Patterson NJ, Plenge RM, Weinblatt ME, Shadick NA, Reich D. Principal components analysis corrects for stratification in genome-wide association studies. Nat Genet. 2006;38: 904–909.

13. Alexander DH, Novembre J, Lange K. Fast model-based estimation of ancestry in unrelated individuals. Genome Res. 2009;19: 1655–1664.

14. Delaneau O, Marchini J, Zagury J-F. A linear complexity phasing method for thousands of genomes. Nat Methods. 2011;9: 179–181.

15. Lawson DJ, Hellenthal G, Myers S, Falush D. Inference of population structure using dense haplotype data. PLoS Genet. 2012;8: e1002453.

16. Hellenthal G, Busby GBJ, Band G, Wilson JF, Capelli C, Falush D, et al. A genetic atlas of human admixture history. Science. 2014;343: 747–751.

17. Reich D, Price AL, Patterson N. Principal component analysis of genetic data. Nat Genet. 2008;40: 491–492.

18. Patterson N, Moorjani P, Luo Y, Mallick S, Rohland N, Zhan Y, et al. Ancient admixture in human history. Genetics. 2012;192: 1065–1093.
