# Supplementary figures and images for: Genetic signatures of gene flow and malaria-driven natural selection in sub-Saharan populations of the "endemic Burkitt Lymphoma belt"
Source: PLoS Genet. 2019 Mar 8;15(3):e1008027. doi: 10.1371/journal.pgen.1008027 (PMC6426263; doi:10.1371/journal.pgen.1008027)

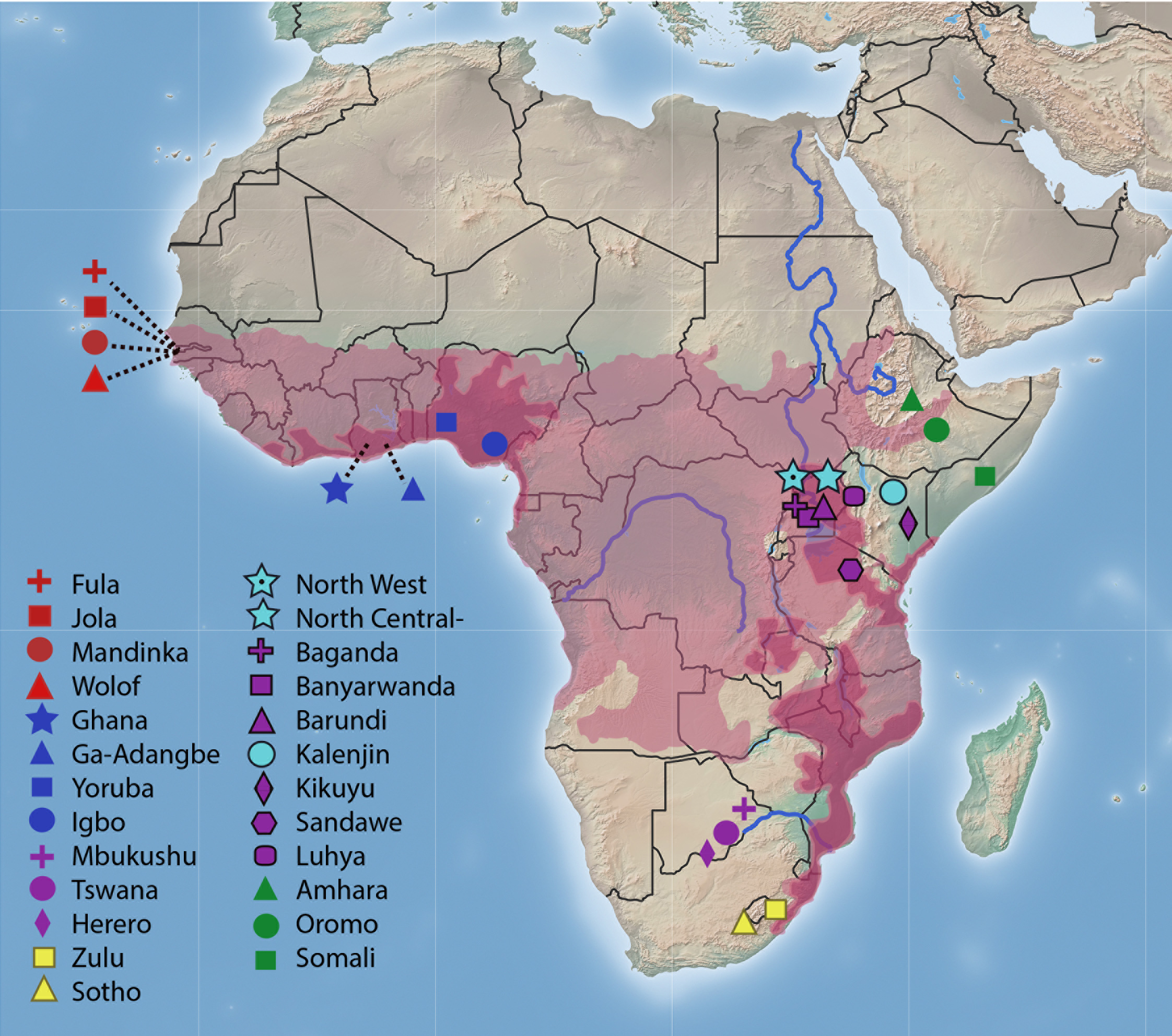

Supplement: S1 Fig — The eBL belt is shown in red shade and the incidence of eBL is denoted by the red color intensity. (TIF) [file pgen.1008027.s002.tif]

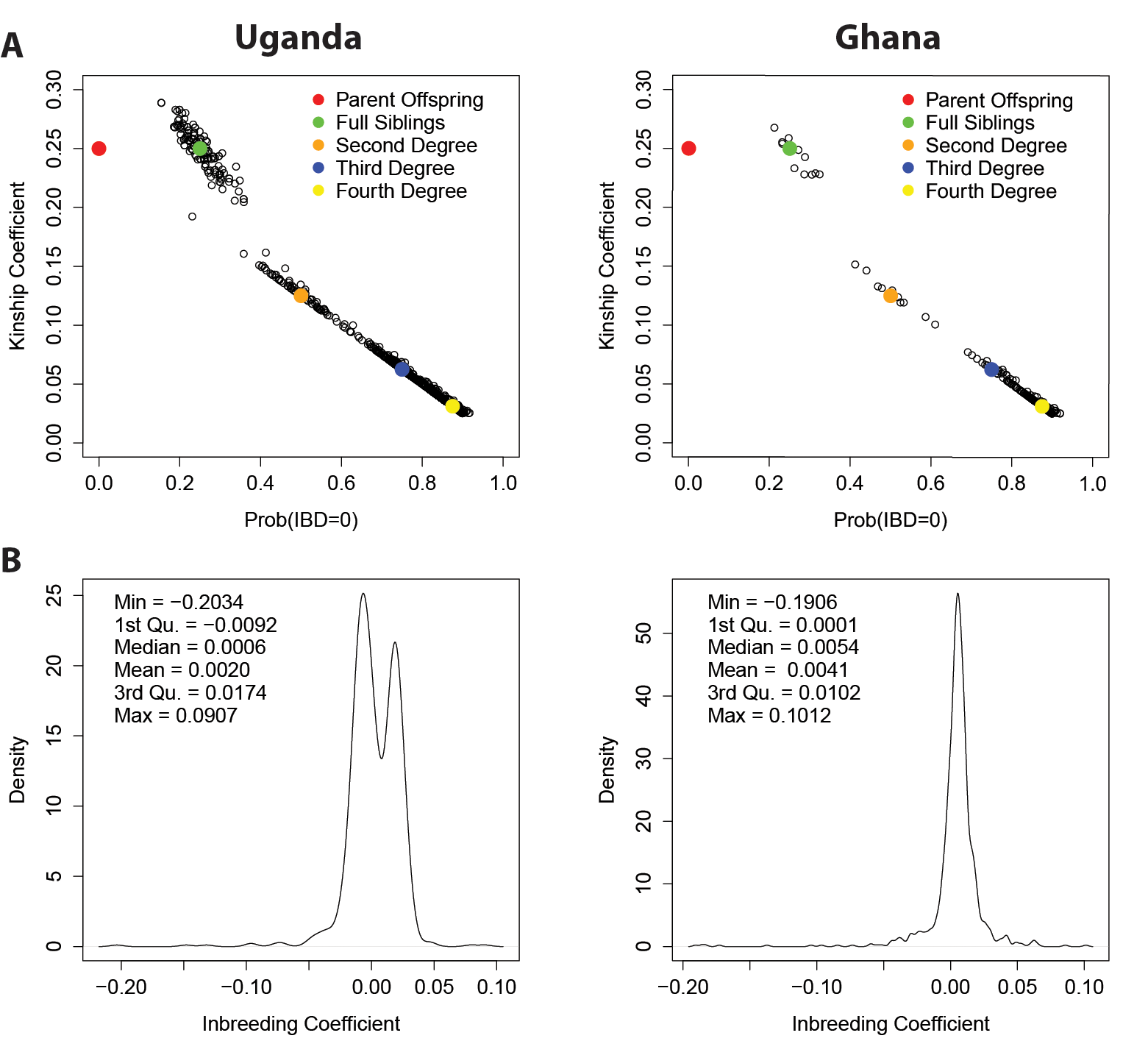

Supplement: S2 Fig — (A) Kinship coefficients (Φij) estimates by the probabilities of IBD = 0 estimates for all pairs of individuals. The colored dots are the theoretical relatedness degree probabilities of Φij and IBD = 0. (B) The distribution of individual inbreeding coefficients estimated for all individuals. (TIF) [file pgen.1008027.s003.tif]

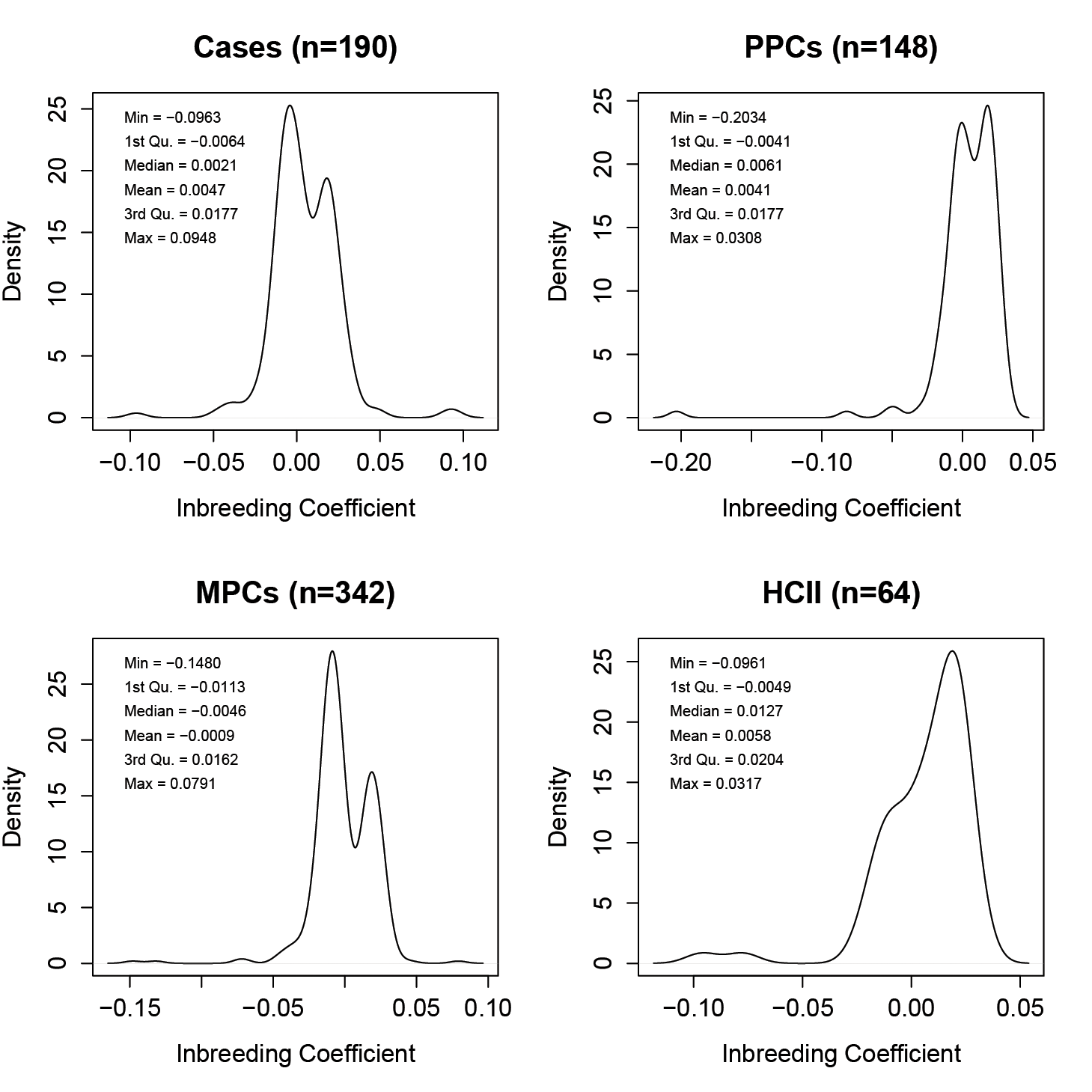

Supplement: S3 Fig — (TIF) [file pgen.1008027.s004.tif]

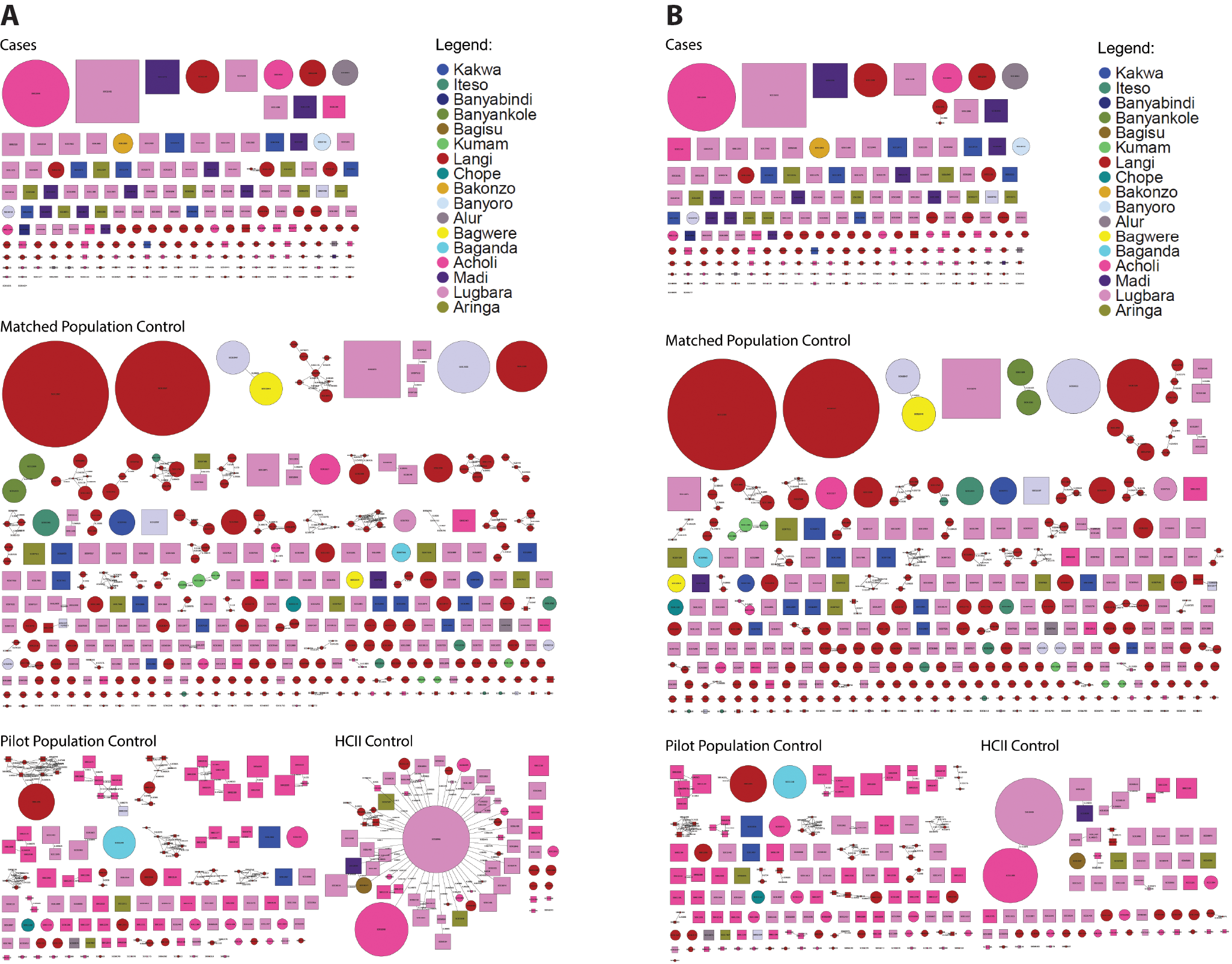

Supplement: S4 Fig — We represented in the same image the individual inbreeding coefficient and the pairwise kinship coefficient (Φij) that represents the relatedness among the individuals. In this network, the nodes are the individuals and the edges are kinship relationships between individuals. Here, we linked only pairs of individuals with Φij ≥ 0.06 (A) or ≥ 0.1 (B), which means we consider as related only individuals with relatedness ≥ third or second degree, respectively. The size of nodes is proportional to the absolute value of individual inbreeding and the shape of the node serves to signal whether inbreeding is positive (square) or negative (circle). The colors of the nodes represent the Uganda individual’s tribe (S1A Table). We represented only the samples with proportion of identity by descent (Plink PI_HAT) > 0.05. (TIF) [file pgen.1008027.s005.tif]

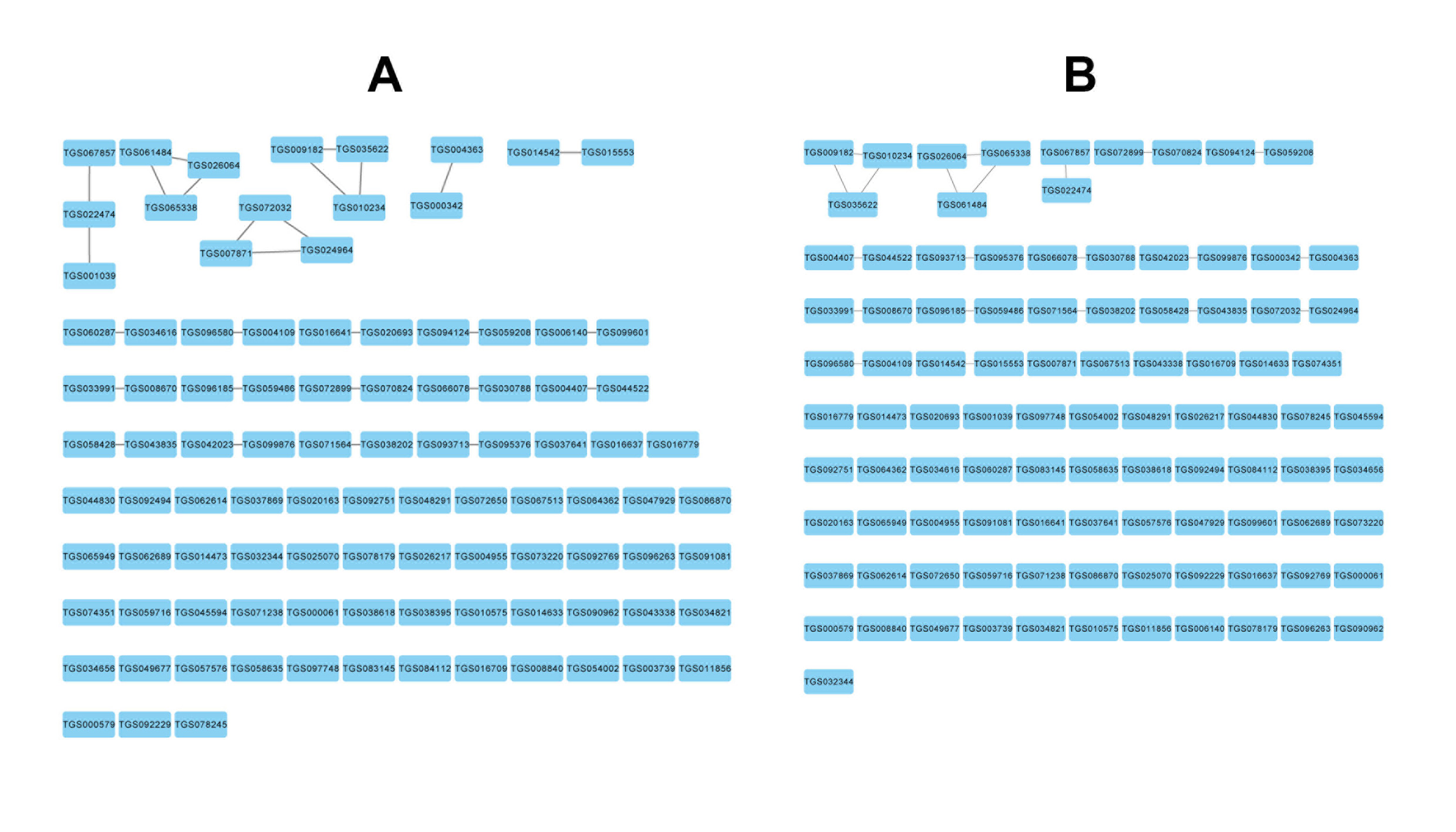

Supplement: S5 Fig — In each network, the nodes are the individuals and the edges are kinship relationships between individuals. We represented only the samples with proportion of identity by descent (Plink PI_HAT) > 0.05. (TIF) [file pgen.1008027.s006.tif]

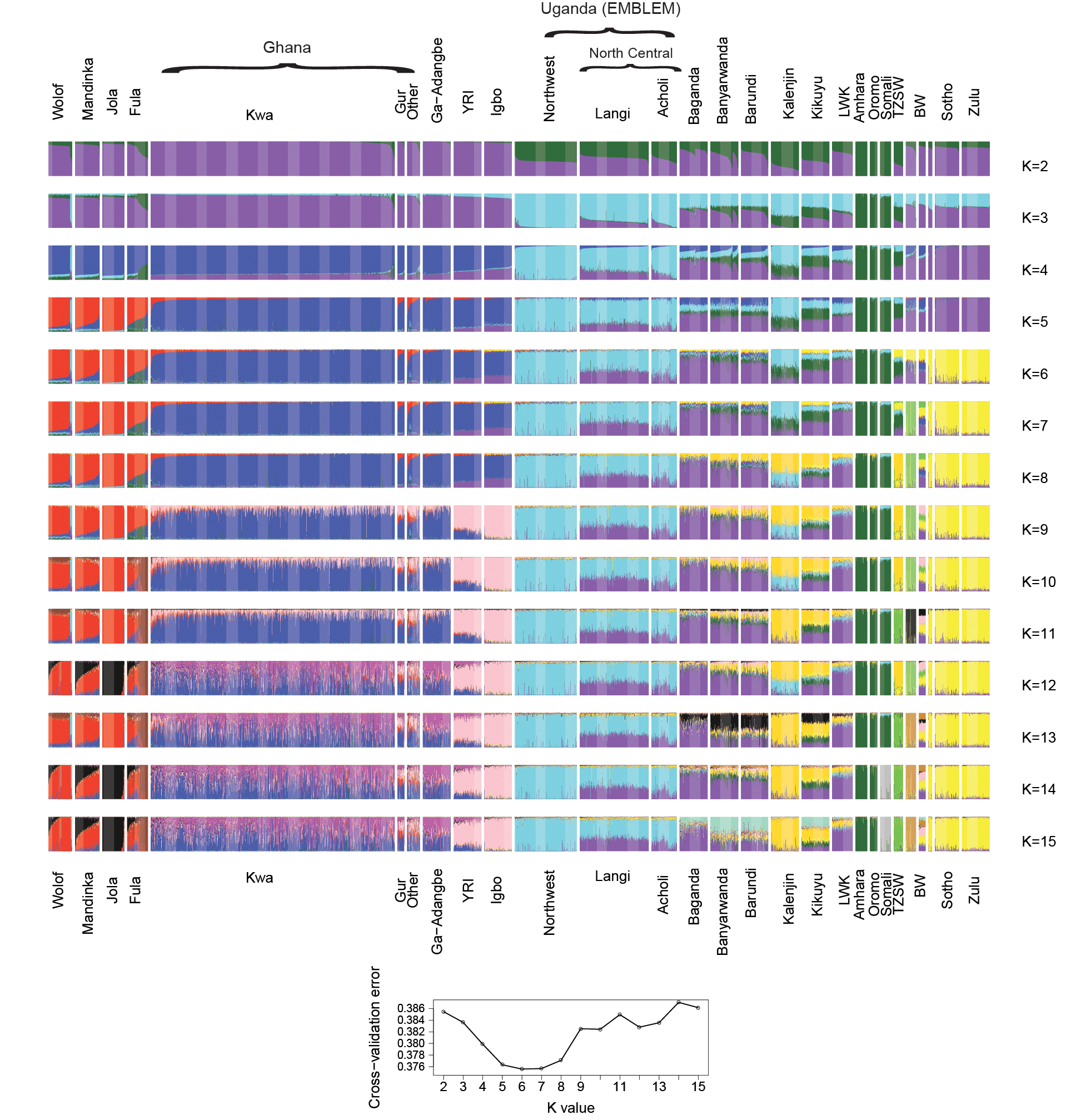

Supplement: S6 Fig — (Top) The proportions of individual ancestry values were calculated using ADMIXTURE unsupervised mode with the number of ancestral K = 2 to K = 15. (Bottom) ADMIXTURE cross-validation errors as a function of K. (TIF) [file pgen.1008027.s007.tif]

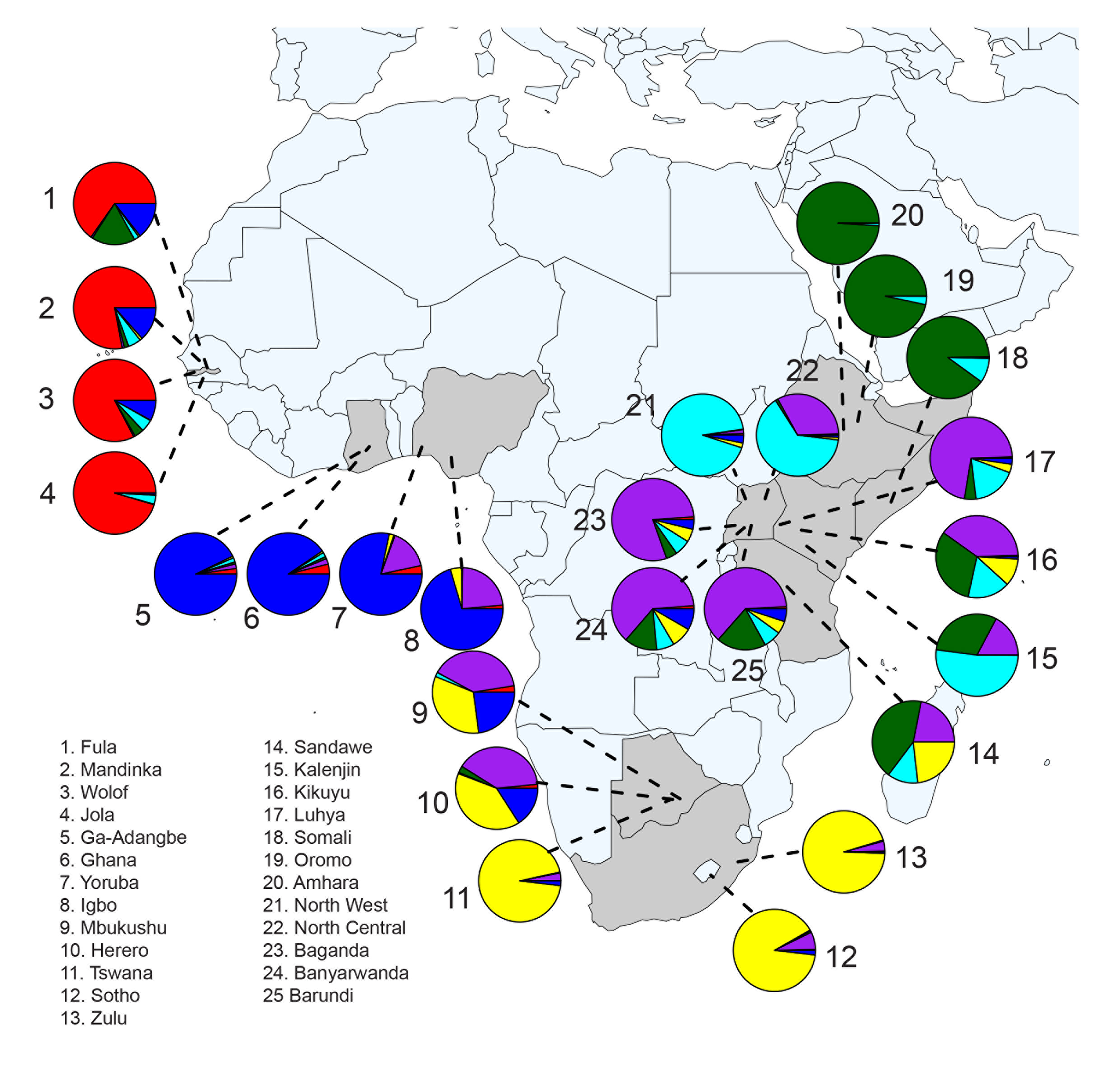

Supplement: S7 Fig — The populations are: Ghana; Uganda North West; Uganda North Central; and three populations from Uganda South West (Baganda, Barundi, and Banyarwanda). The Pan-African populations are from West Africa (Fula, Mandika, Wolof, and Jola), West Central Africa (Ga-Adangbe, Yoruba, and Igbo), Southern Africa (Mbukushu, Herero, Tswana, Zulu, and Sotho), Horn of Africa (Amhara, Oromo, and Somali) and Great Lakes Africa (Luhya, Kalenjin, and Kikuyu). (TIF) [file pgen.1008027.s008.tif]

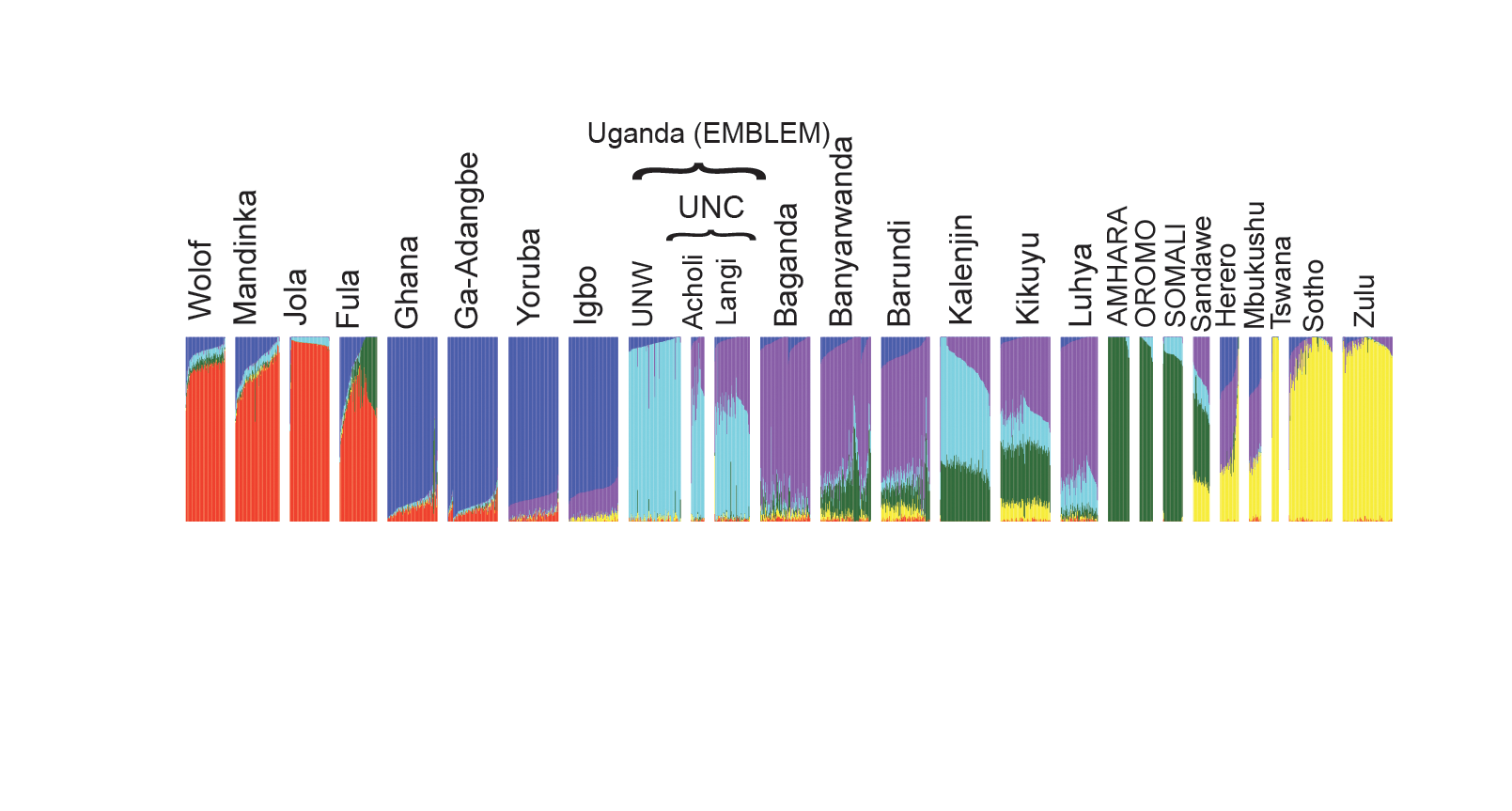

Supplement: S8 Fig — (TIF) [file pgen.1008027.s009.tif]

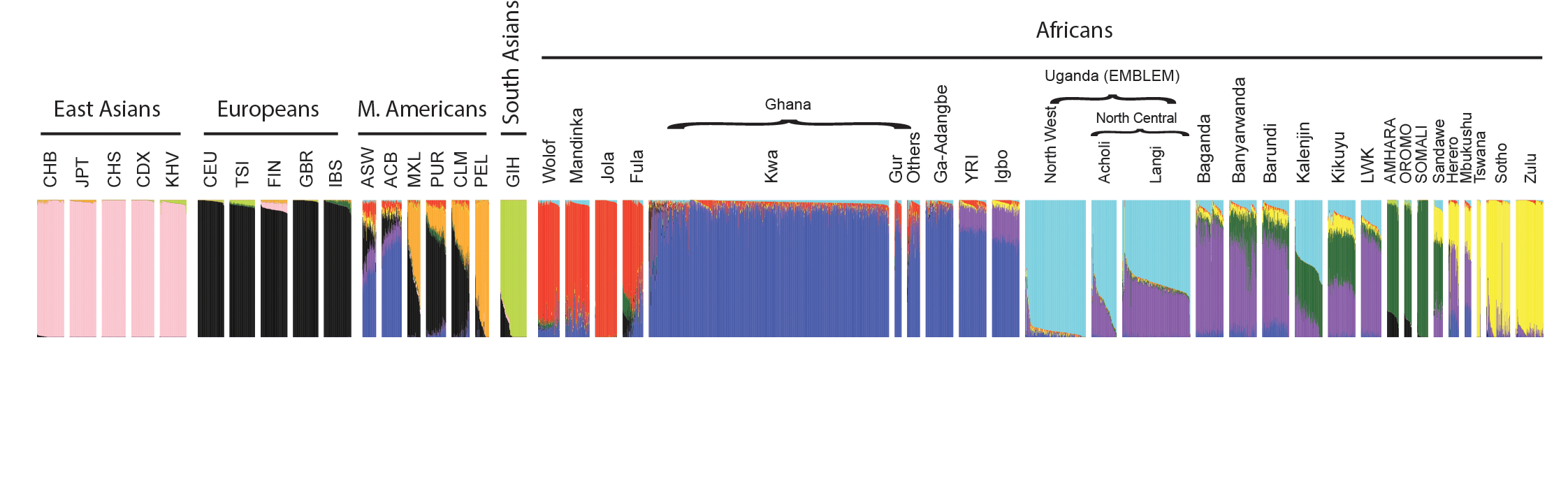

Supplement: S9 Fig — We represented the unsupervised ADMIXTURE analysis with the number of ancestral clusters K = 10. This K captured the six African clusters represented in Fig 1C, and East Asian (pink), and South Asian (light green) Asian, European (black) and Native American (orange) ancestral clusters. ASW—Americans of African Ancestry in SW USA; MXL—Mexican Ancestry from Los Angeles USA; PUR—Puerto Ricans from Puerto Rico; ACB—African Caribbeans in Barbados; CLM—Colombians from Medellin, Colombia; PEL—Peruvians from Lima, Peru; KHV—Kinh in Ho Chi Minh City, Vietnam; JPT—Japanese in Tokyo, Japan; CDX—Chinese Dai in Xishuangbanna, China; CHB—Han Chinese in Beijing, China; CHS—Southern Han Chinese; GIH—Gujarati Indian from Houston, Texas. (TIF) [file pgen.1008027.s010.tif]

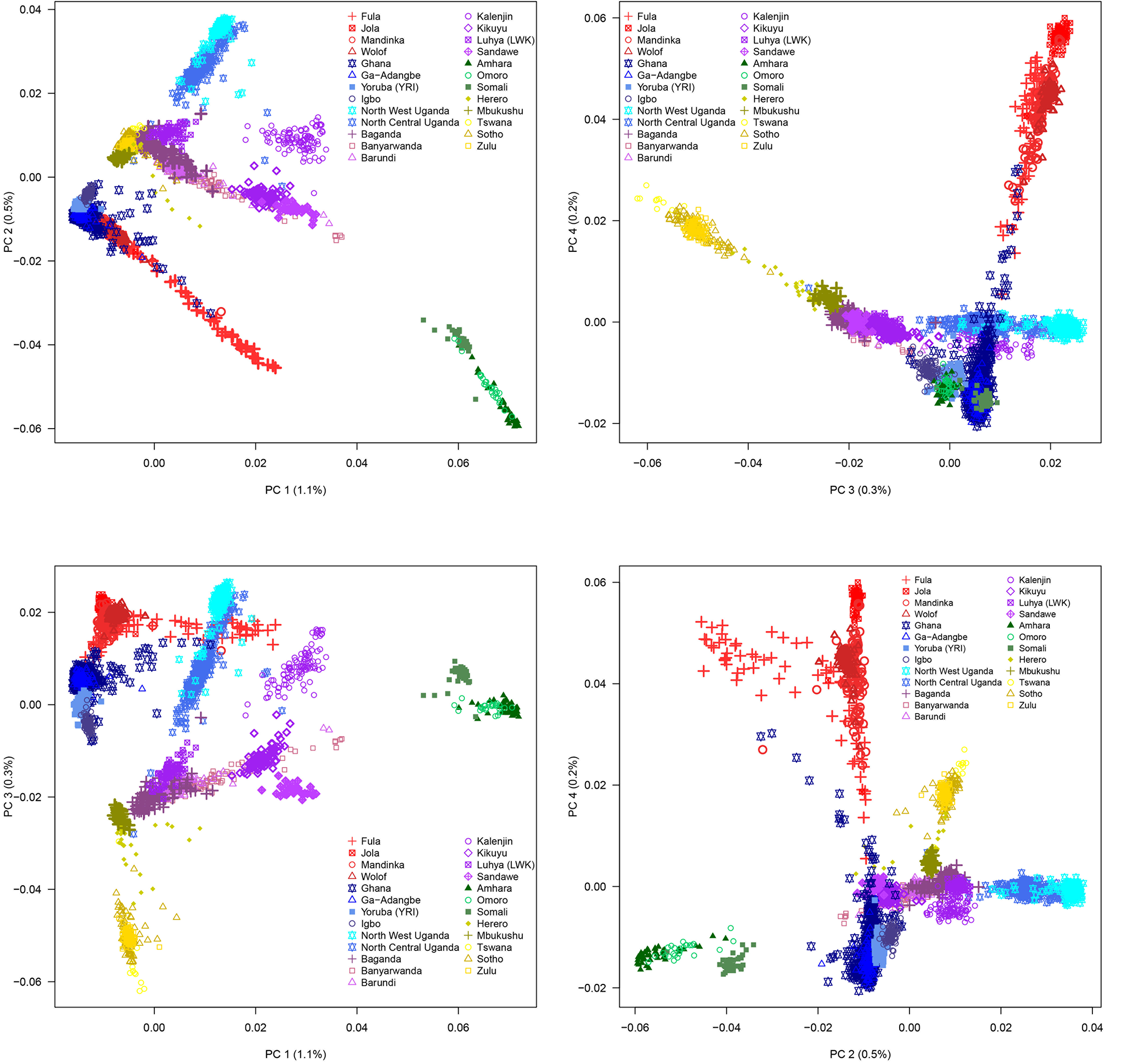

Supplement: S10 Fig — We compared the following PC combinations: PC1 vs PC2, PC3 vs PC4, PC1 vs PC3 and PC2 vs PC4. (TIF) [file pgen.1008027.s011.tif]

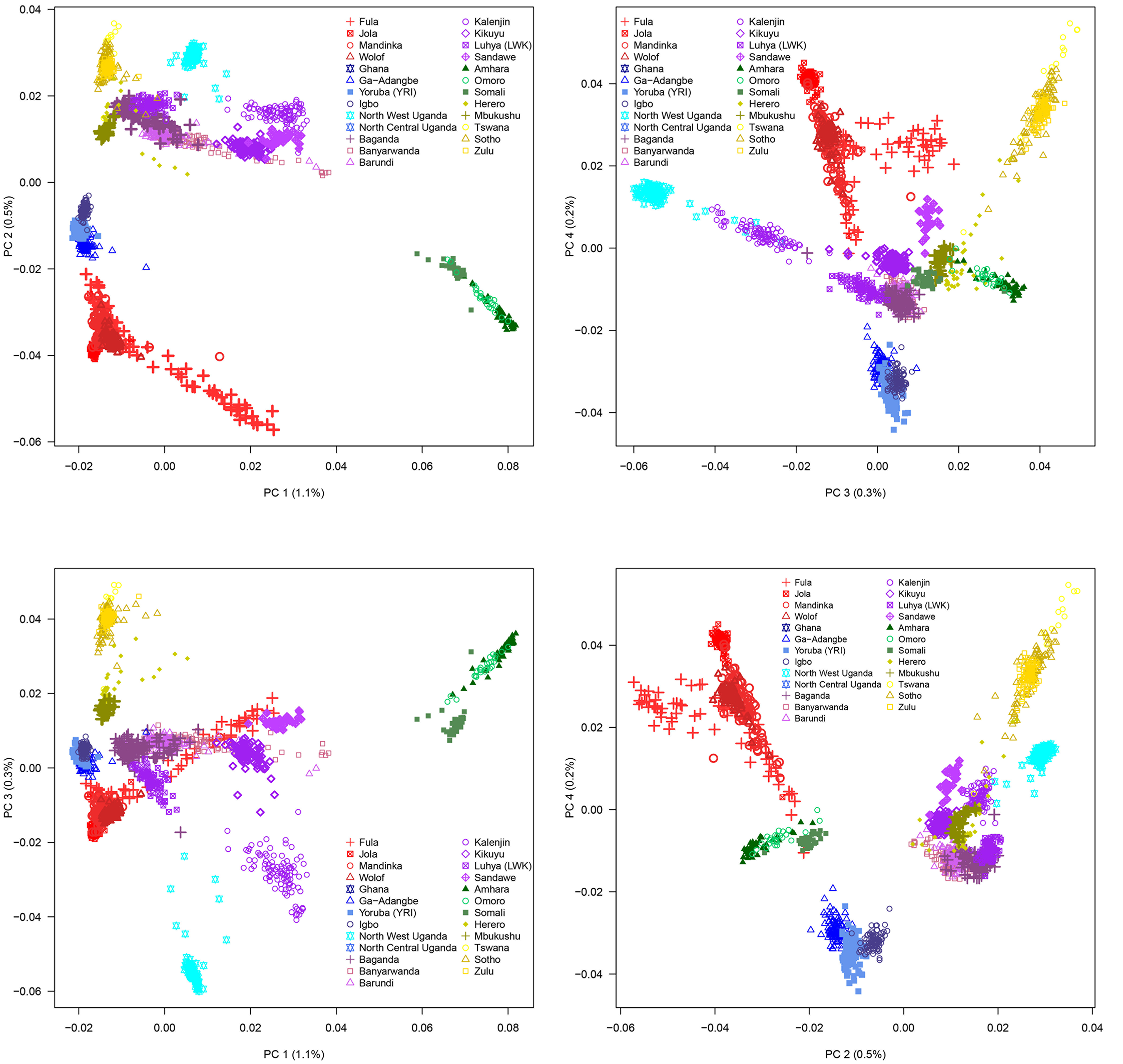

Supplement: S11 Fig — We compared the following PC combinations: PC1 vs PC2, PC3 vs PC4, PC1 vs PC3 and PC2 vs PC4. (TIF) [file pgen.1008027.s012.tif]

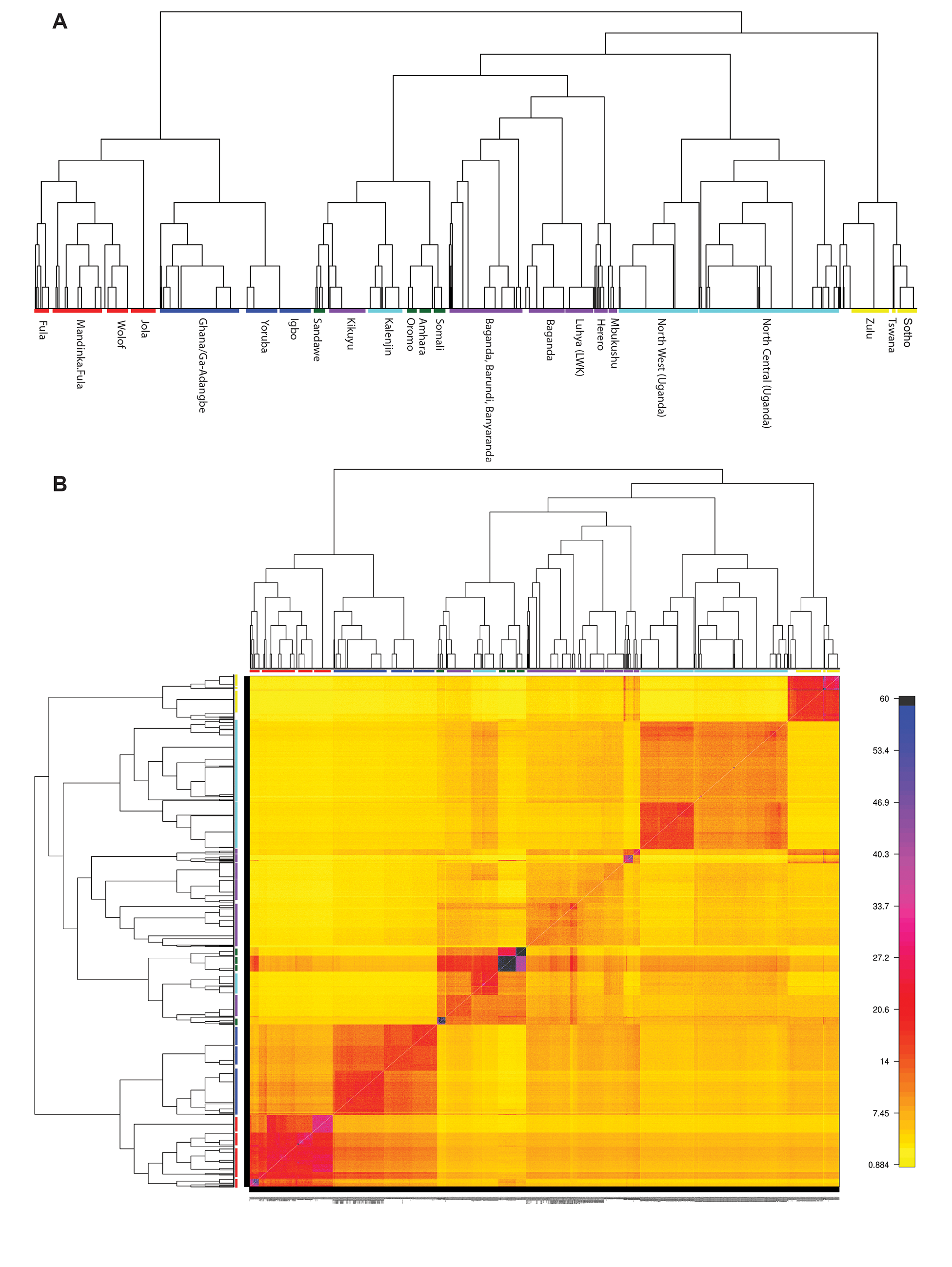

Supplement: S12 Fig — (A) fineSTRUCTURE tree and (B) heatmap of the length of the chunks shared by individuals. Each row of the heatmap represent a copyvector of a recipient individual and each column represent the proportions of haplotypes that a donor shares with a recipient. Dark regions of the heatmap represent the long haplotype segments shared between individuals. Dark regions outside on the diagonal indicate more recent gene flow events. We highlight the inferred clusters using the colors of the ADMIXTURE ancestries. (TIF) [file pgen.1008027.s013.tif]

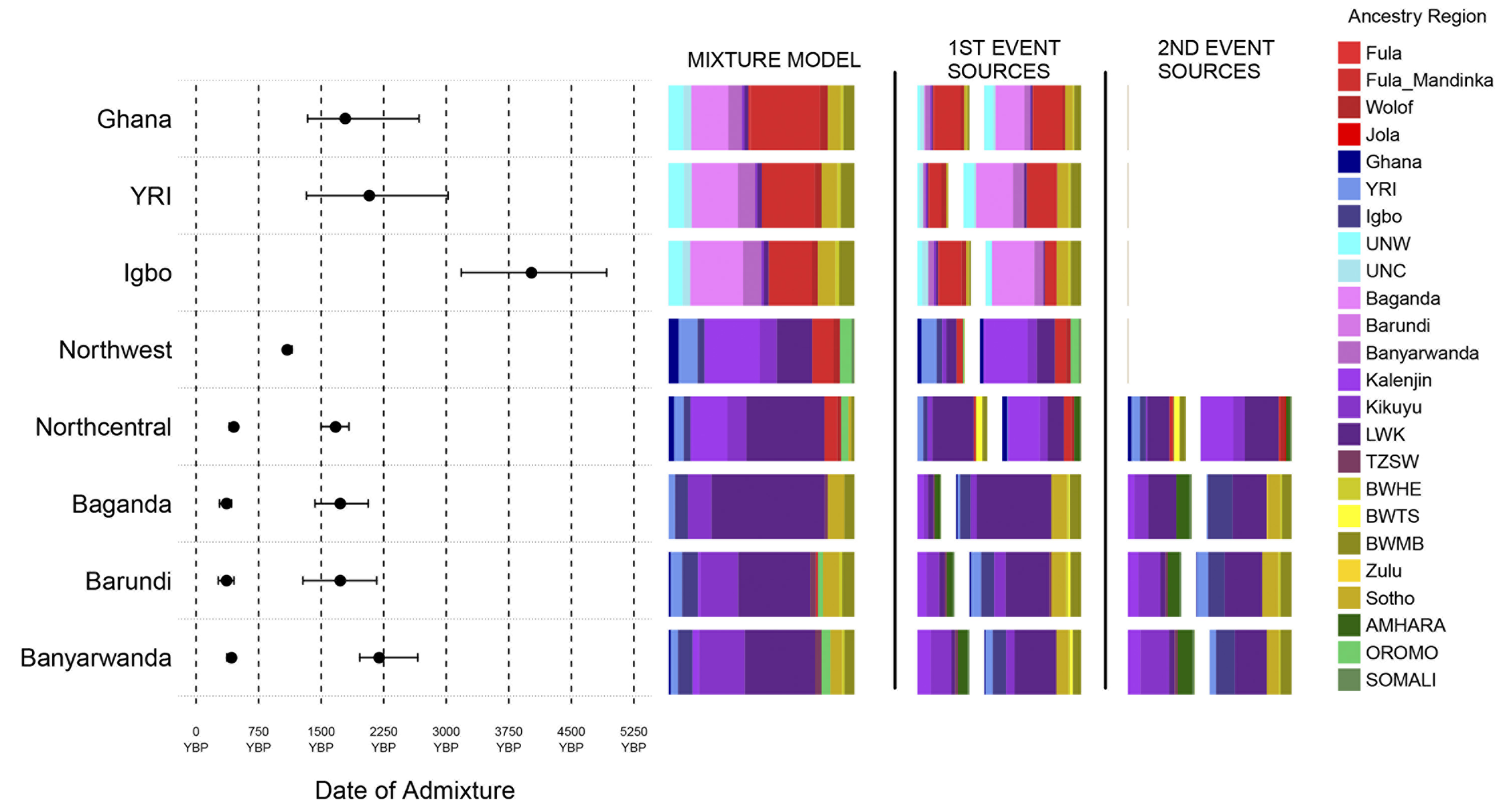

Supplement: S13 Fig — Ancestry profiles and admixture dynamics were inferred using non-local donors. Donor populations were selected based on fineSTRUCTURE results. In the mixture model and event sources, the bars show the contribution of each African population to the recipient populations. The plot on the left represents the most likely estimated admixture dates inferred by GLOBETROTTER. The plot shows two admixture events, except in the West Africans and North West Uganda where only one admixture event was found. Inferred date(s) and 95% CIs are represented by the dots and horizontal lines in the graph. Bars corresponding to the event sources represent the inferred admixing sources for each estimated admixture event and the proportion of contribution of the African donor populations. (TIF) [file pgen.1008027.s014.tif]

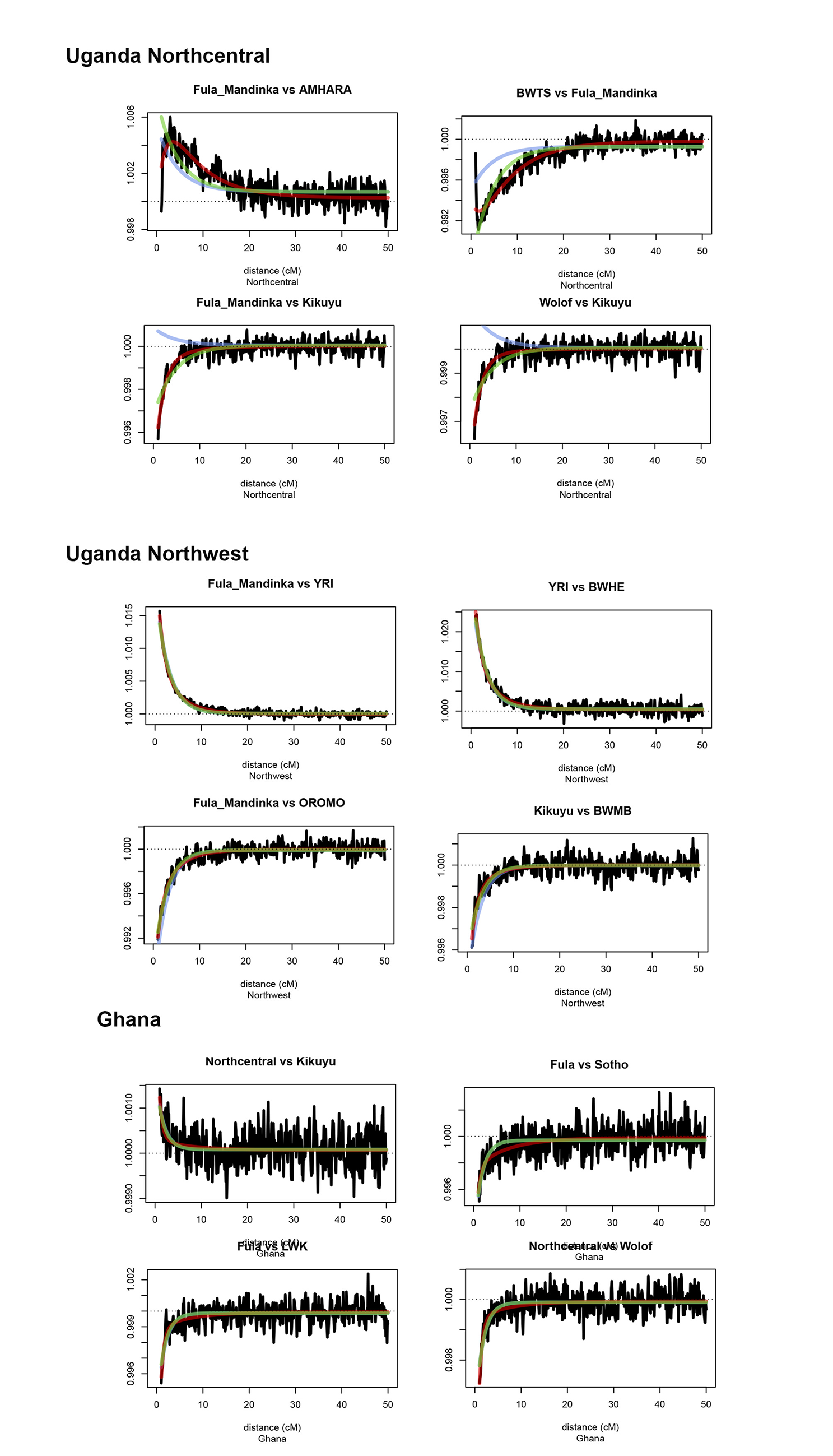

Supplement: S14 Fig — These curves are informative for date estimation and the genetic composition of the sources of the admixture event. Each curve describes the probability to find two chunks of two donor populations along the genome of the target population. Curves with decreasing probability indicate that the two donor populations describe the genetic composition of one source population. Increasing probability indicates that the two donors could describe different sources. One date admixture (North West and Ghana) is characterized by a uniform curve that decreases or increases its probability. On the other hand, multiple date admixture (North Central) is characterized by a curve that changes its behavior, for example, from increasing (indicating different donor for the earlier event) to decreasing (both donor populations describe one source for the recent event). (TIF) [file pgen.1008027.s015.tif]

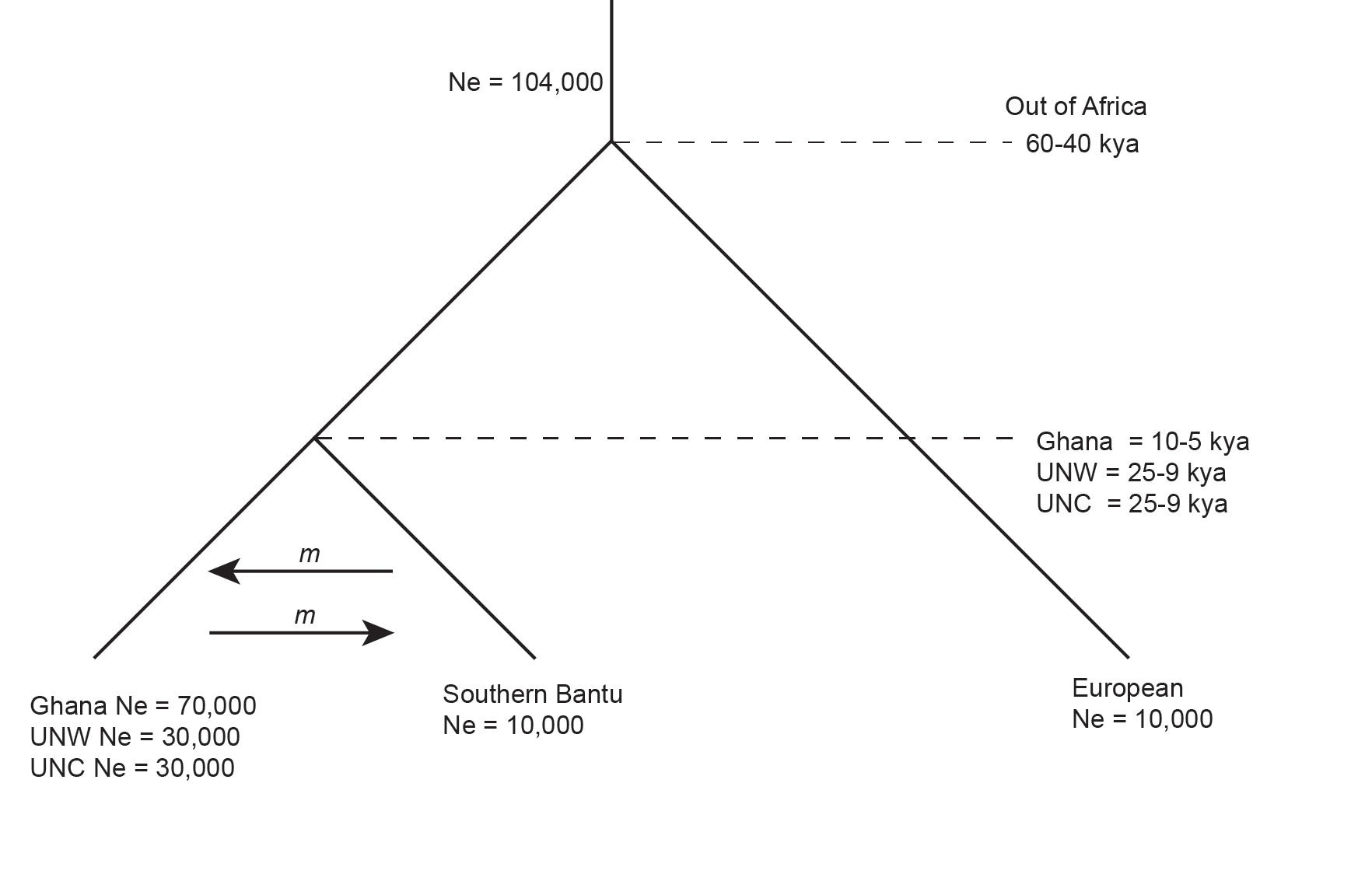

Supplement: S15 Fig — Ne = effective population size, kya = thousand years ago and m = migration rate. We used the migration rates following the current ancestry profile estimated by ADMIXTURE, as 4Nemij, where 4Ne is the population effective size and mij the fraction of population i that is made up of migrants from population j. The simulated genotypes were obtained by the ms program [76] with the following command line: ms NTotalPop 10000 –s 1 –t 0.01 –I 3 NPop1 NPop2 NPop3 –eg 0.002 1 gPop1 at time1 –em 0.002 1 3 NMigrantsij−em 0.002 3 1 NMigrantsji -ej 0.025 3 1 –en 0.06 1 gPop1 at time2 –en 0.06 1 gPop2 at time2 –ej 0.06 2 1 –en 0.1 1 gPop1 at time3. (TIF) [file pgen.1008027.s016.tif]

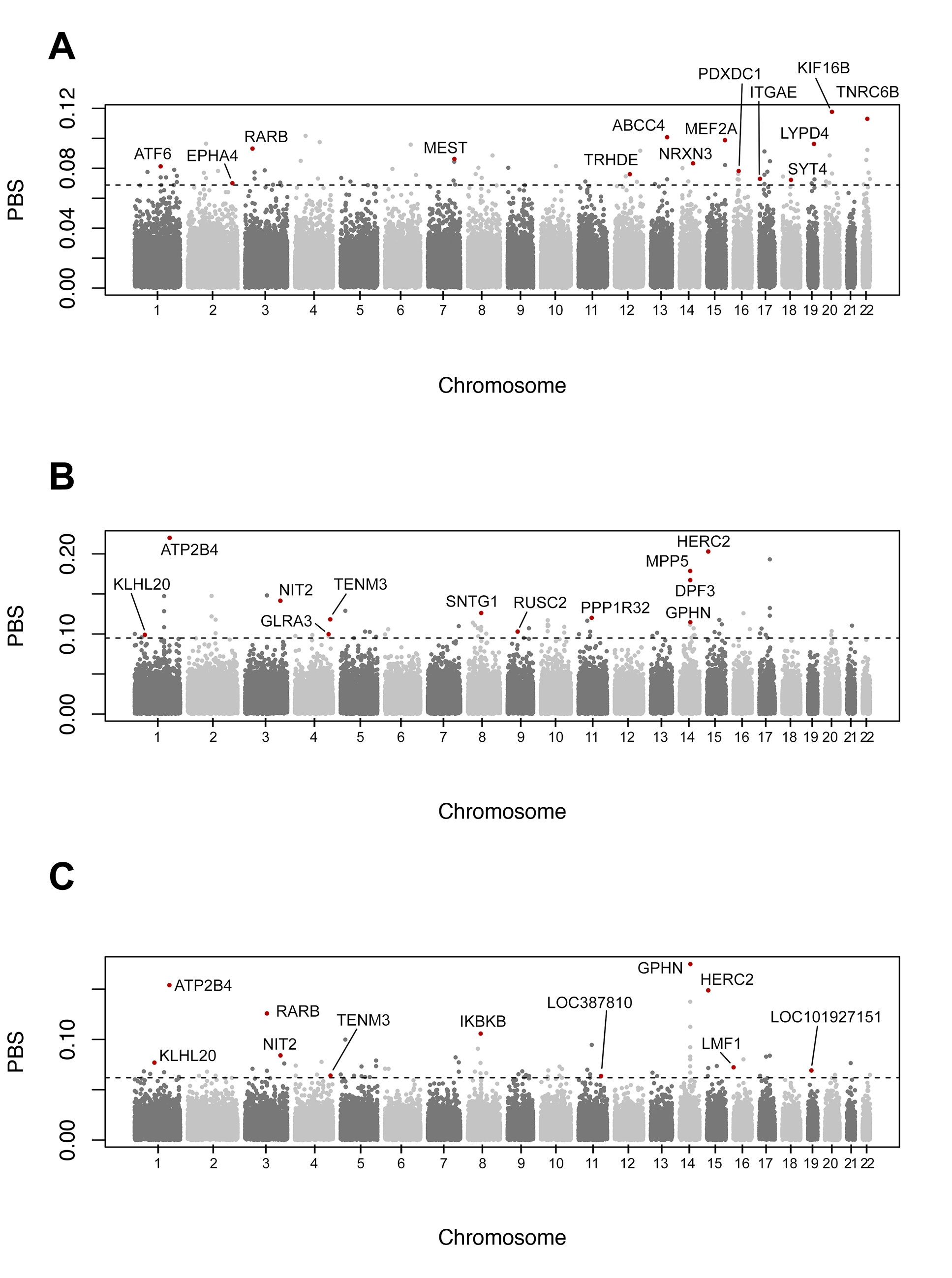

Supplement: S16 Fig — The dotted line demarcates the 99.9th percentile. The red dots represent genes that also have a selection signal by the xpEHH test (>2). (TIF) [file pgen.1008027.s017.tif]

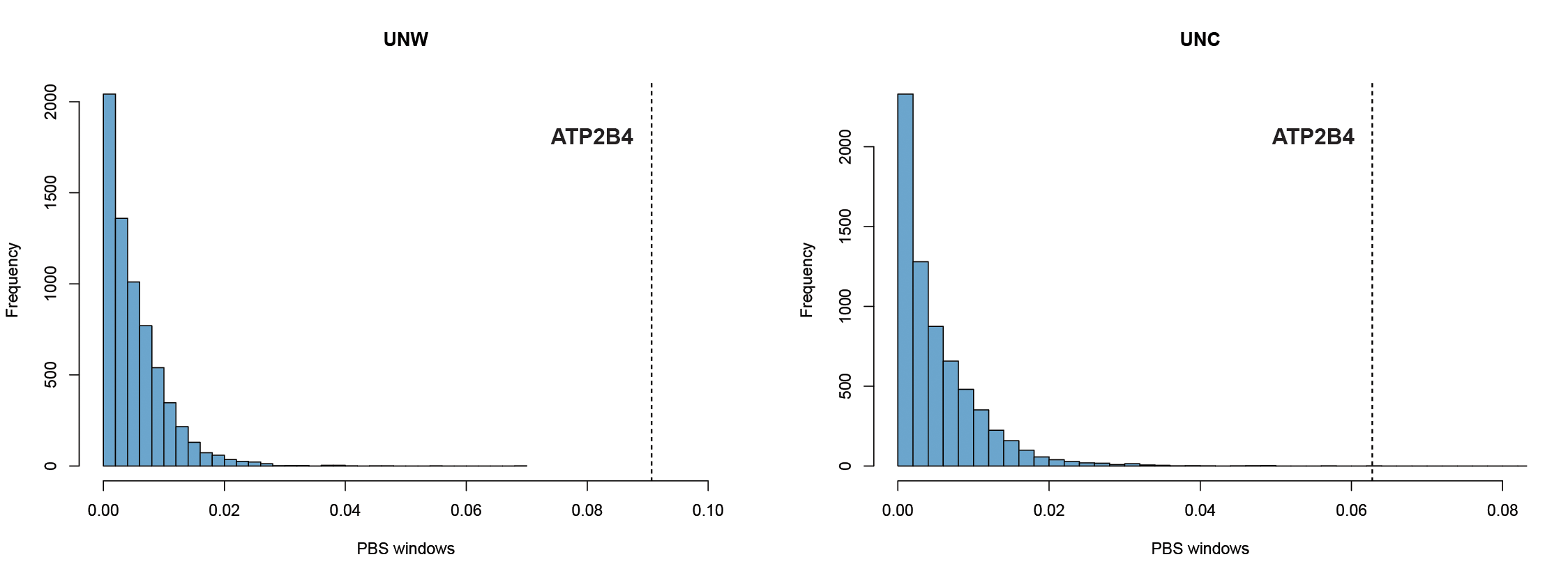

Supplement: S17 Fig — PBS neutral values were generated by 10,000 simulations of plausible neutral demographic models (S15 Fig) for UNW and UNC populations respectively. (TIF) [file pgen.1008027.s018.tif]

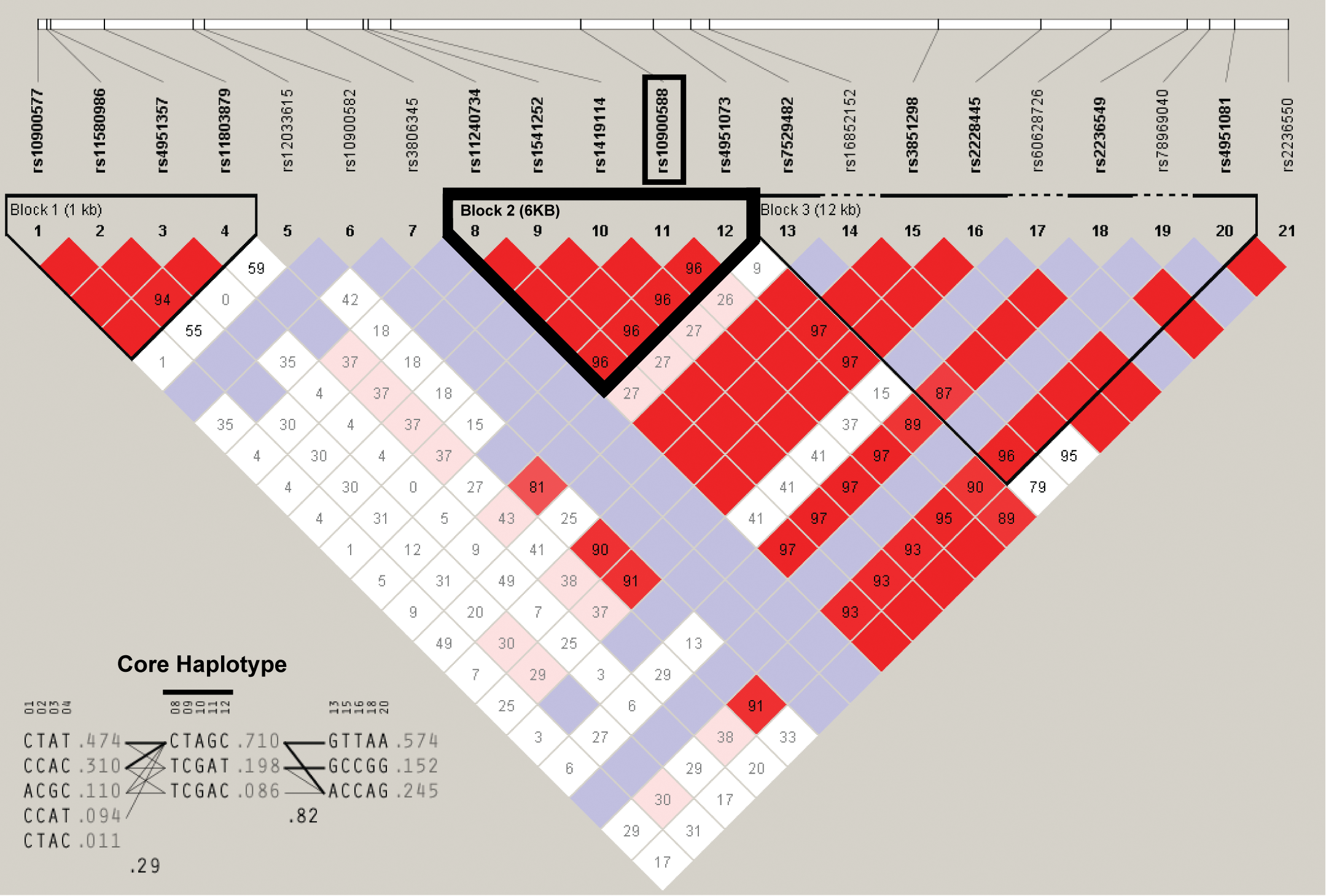

Supplement: S18 Fig — We presented the LD table of the Uganda North West (UNW) population that showed the highest signal of selection. (TIF) [file pgen.1008027.s019.tif]
